# GPU-Accelerated Virtual Screening and Molecular Dynamics Simulations for Identification of Novel DPP‑4 Inhibitors

**DOI:** 10.1021/acsomega.5c08231

**Published:** 2026-01-21

**Authors:** Nathaly Vasquez-Martínez, Jonathan Trapala, Laura I. Álvarez-Añorve, Rodolfo A. Lizárraga-Valadez, Martín González-Andrade, Alejandro Sosa-Peinado

**Affiliations:** † Departamento de Bioquímica, Facultad de Medicina, 61589 Universidad Nacional Autónoma de México, Ciudad de México 04510, México; ‡ Departamento de Ingeniería Celular y Biocatálisis, Instituto de Biotecnología, Universidad Nacional Autónoma de México, Cuernavaca 62210, México

## Abstract

Inhibition of dipeptidyl peptidase 4 (DPP-4) is a crucial therapeutic strategy for the management of type 2 diabetes mellitus (T2DM). However, current inhibitors often exhibit unwanted toxicity, underscoring the need to discover novel, selective, and safer alternatives. This study employs an integrated computational pipeline to accelerate the identification of new DPP-4 inhibitor candidates. To that effect, GPU-accelerated molecular docking of 30,699 bioactive PubChem compounds was combined with molecular dynamics (MD) simulations and membrane permeability analyses. A workflow that systematically filters candidates was presented based on the score binding predicted by Uni-Dock. Subsequently, the stability of 32 promising protein–ligand systems was assessed using 100 ns MD trajectories, confirming their stable binding to the DPP-4 active site. Compounds EPZ005687, OSU-03012, and bemcentinib showed higher binding affinity and more favorable interactions within pockets S1, S2, S1′, S2′, and S2 ′ than the FDA-approved reference drugs like alogliptin, based on MM-GBSA calculations. To assess the therapeutic viability of the candidates, their cellular absorption potential was also investigated. Permeability (free energy of transfer profile) and interactions were calculated via Umbrella Sampling and long-time MD across a physiologically relevant enterocyte membrane model. The results revealed that EPZ005687, OSU-03012, and bemcentinib exhibited better permeation characteristics than alogliptin. This combined evidence of high target affinity and enhanced cellular permeability strongly suggests these compounds are up-and-coming antidiabetic agents. These findings demonstrate the efficacy of this integrated computational strategy, along with the utilization of rigorously filtered public databases, for accelerating the discovery of safer and more effective antidiabetic treatments.

## Graphical abstract

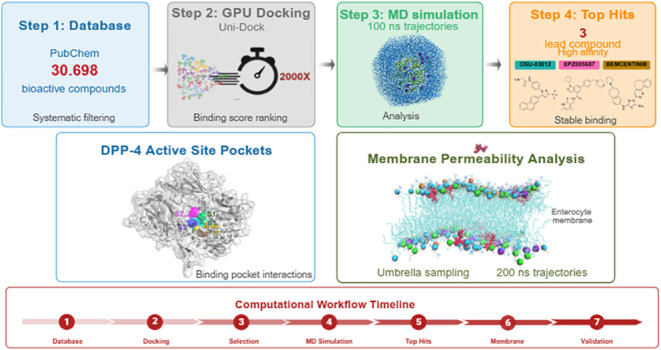

## 1 Introduction

Diabetes mellitus (DM) represents a significant global public health challenge, affecting around 589 million adults aged 20 to 79 and causing 3.4 million deaths in 2024, with prevalence anticipated to reach 853 million by 2050.^1^ This group of chronic metabolic diseases is characterized by hyperglycemia resulting from impaired β-cells function or reduced tissue insulin responsiveness.^2^ Diabetes predisposes individuals to infections and diverse long-term complications, including nephropathy, neuropathy, and cardiovascular disease, which are associated with increased morbidity and mortality rates. While primary treatment of DM focuses on maintaining glycosylated hemoglobin (HbA1c) below <7% (53 mmol/mol) through oral hypoglycemic agents,^3,4^ this approach often fails to eliminate the long-term risk of complications, highlighting the need for novel therapeutic targets.^5^

DPP-4 (EC 3.4.14.5), an endogenous serine exopeptidase, is a proven molecular target for the treatment of type 2 diabetes (T2DM). Its inhibition enhances glucose-induced insulin secretion through incretin hormone pathways (Figure 1), establishing its therapeutic potential.^6,7^

**Figure 1:**
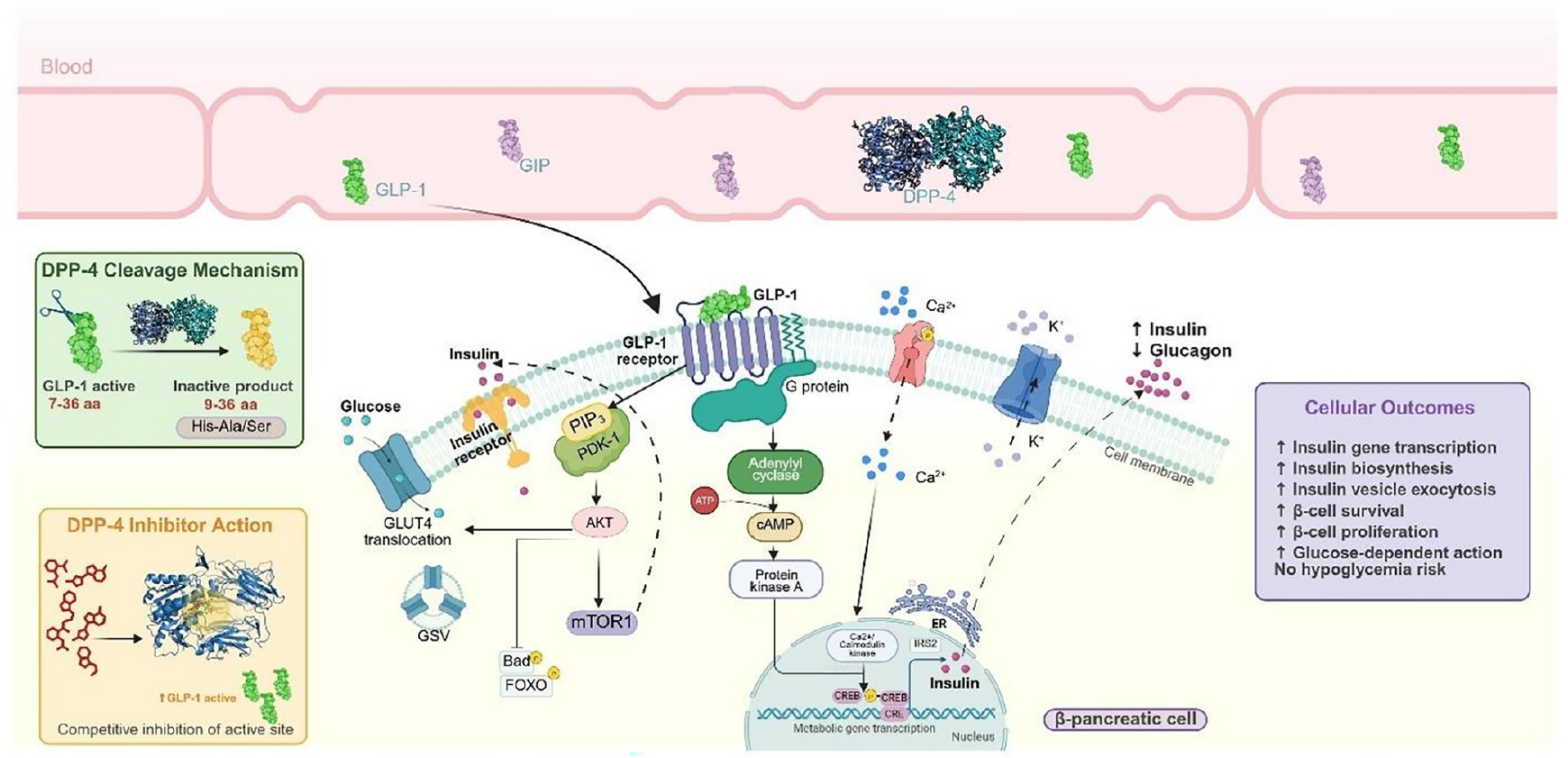
DPP-4 cellular mechanism of action. The incretins GLP-1 and GIP circulate in the blood as substrates for DPP-4. This enzyme cleaves active incretins, converting them into inactive forms that cannot bind to their receptors. A DPP-4 inhibitor blocks the enzyme’s active site, preventing degradation and allowing the incretin to remain active. Active GLP-1 then binds to its receptor (GLP-1R) on the pancreatic β-cell membrane, activating a G-protein signaling pathway that regulates gene transcription, insulin biosynthesis, and cell survival. In parallel, insulin binding to its receptor activates the PI3K/AKT pathway, which converges with GLP-1 signaling to amplify the cellular response, leading to multiple metabolic benefits.^8^

Since the first DPP-4 inhibitor (DPP-4*i*) received US Food and Drug Administration (FDA) approval in 2006, more than a dozen synthetic gliptins have been developed,^9^ classified based on their anchoring to the enzyme catalytic pocket (S1, S1′, S2, S2′, and S2 extensive).^10–12^ FDA-approved gliptins are administered orally, with bioavailability ranging from 30% to 100%.^13^ Despite promising pleiotropic effects—improved glycemic control by enhanced insulin secretion and HbA1c reduction, and organ-protective benefits,^14–17^ pharmacovigilance studies have identified significant adverse events associated with its administration. These frequently include nonspecific gastrointestinal inflammation, acute pancreatitis, hypersensitivity, angioedema, severe cutaneous adverse reactions, and anaphylactic reactions,^18^ underscoring the need for new, safe, and selective inhibitors.

In drug discovery, combining docking with molecular dynamics (MD) is a valuable *in silico* strategy for a comprehensive understanding of target-drug interactions.^19–23^ This approach allows the study of complex stability and dynamic behavior, thereby optimizing the entire discovery pipeline.^24–26^ Notably, implementation of multiple graphics processing units (GPUs) has transformed structure-based virtual screening workflows by dramatically accelerating the evaluation of large compound libraries against particular protein targets. Beyond simple speed enhancement, GPU acceleration provides critical advantages, including enhanced parallel processing for the simultaneous evaluation of multiple binding conformations, improved resource efficiency enabling thorough conformational sampling, and superior scalability for exploring larger chemical spaces.^27–30^ Today, natural compounds constitute a promising source of lead molecules for therapeutic screening, as they have greater chemical diversity and are safer than manufactured drugs.^31^ Their intrinsic structural diversity and relative bioactivity offer drug discovery advantages by promoting selective and specific protein target interactions.^32^

Once promising compounds are identified, a significant limitation is their low permeability through lipid membranes, which directly affects target-site access, bioavailability, and therapeutic potential. Poor membrane permeability frequently leads to the failure of otherwise promising drug candidates during preclinical and clinical development phases.^33^ To address this limitation, computational methods for predicting passive transport have gained significant traction in drug discovery pipelines as cost-effective screening tools that identify permeability issues early in development, before advancing to costly experimental phases.^34^

This study presents a thorough computational investigation aimed at identifying novel DPP-4*i* candidates that exhibit both potent target binding and enhanced membrane permeability, thereby overcoming current therapy limitations. The main aim was to establish the theoretical foundation for natural compounds-based DPP-4*i* discovery, driven by the hypothesis that a computational pipeline combining rational database selection with GPU-accelerated virtual screening could efficiently identify promising candidates. Accordingly, ∼30,000 natural compounds from PubChem, selected for reported nanomolar and micromolar bioactivities against diverse therapeutic targets, were subjected to GPU-accelerated docking using Uni-Dock.^30^ Following initial screening, the promising candidates were then rigorously refined based on theoretical binding energy, reported toxicity profile, and ensuring no prior DPP-4 or antidiabetic activity. The energetic stability, protein flexibility in the presence of ligands, and protein–ligand interactions of selected candidates were then assessed in a dynamic environment through MD. Additionally, we evaluated the potential therapeutic effects of EPZ005687 and OSU-03012 by sampling energy profiles and a novel spontaneous passive permeability simulation protocol in an enterocyte lipid bilayer model. The compounds proposed herein represent potential antidiabetic molecules and warrant evaluation *in vitro* and *in vivo* in future studies.

## 2 Materials and Methods

### 2.1 Construction of the Compound Database

Compounds were sourced from the PubChem database (https://pubchem.ncbi.nlm.nih.gov/, accessed February 10, 2025). Initially, a keyword search using the term “*Natural*” compounds was performed. This data set was then refined by applying a chemical vendor filter to remove entries that were not commercially available. To focus on pharmaceutically relevant compounds, the last bioactivity-based filter retained only molecules with demonstrated nanomolar or micromolar activity. These filtered data sets were then subjected to virtual screening using docking.

### 2.2 Protein and Ligand Preparation

The 3D structure of human DPP-4, in complex with an FDA-approved inhibitor, was obtained from the Research Collaboratory for Structural Bioinformatics Protein Data Bank (RCSB PDB; https://www.rcsb.org/) (PDB ID: 2ONC). The structure was prepared for docking using PyMOL Molecular Graphics System, Version 2.5.0 (Schrödinger, Inc., New York, NY, USA).^35^ This preparation involved retaining only the A chain and removing the cocrystallized ligand, ions, crystallographic water molecules, and NAG residues.

For comparative analyses, 3D structures of FDA-approved DPP-4 inhibitors [linagliptin (PubChem CID 10096344), saxagliptin (PubChem CID 11243969), sitagliptin (PubChem CID 4369359), and vildagliptin (PubChem CID 6918537)] were downloaded from the PubChem database. Acetaminophen (PubChem CID: 1983) was used as the negative control. All SDF files were subsequently converted to a *pdbqt* format using Open Babel version 3.1.1 (University of Pittsburgh, Pittsburgh, USA).^36^ This conversion also assigned polar hydrogen atoms and Gasteiger-Marsili charges at pH 7.4, and removed duplicate compounds from the databases.

### 2.3 Molecular Docking

Docking studies were conducted using Uni-Dock, a GPU-accelerated virtual screening program, to identify potential binders to the DPP-4 pocket.^30^ Uni-Dock version 1.1 was configured with CUDA toolkits and an NVIDIA GeForce RTX 4090 graphics card to enable accelerated execution. The docking grid center was generated using AutoDockTools 1.5.7;^37^ referenced to the co-crystal structure of DPP-4 with alogliptin (PDB: 2ONC). The box size was 50 Å in the *x*, *y*, and *z* dimensions, with a spacing grid of 0.375 Å. The center coordinates were −41.83, −18.17, and 15.25 for *x*, *y*, and *z*, respectively. Docking results were reported as free binding energy (kcal/mol). Best conformational states were visualized using PyMOL, and receptor–ligand interactions were generated with Maestro Visualizer v.14.3 (Schrödinger, Inc., New York, NY, USA).

### 2.4 Molecular Dynamics Simulation Studies

#### 2.4.1 Data Selection

Following docking studies, compounds were filtered based on their binding energies, selecting those with the most favorable energies—corresponding to the lowest percentile (<1%) of the energy distribution. Additional exclusion criteria were applied to this group, resulting in a final subset for MD. Antidiabetic drugs (linagliptin, saxagliptin, sitagliptin, vildagliptin, and alogliptin) and acetaminophen as a negative control were also subjected to MD as a reference standard.

#### 2.4.2 Preparation of the System for Molecular Dynamics Simulation

Selected compounds coordinates from the docking were processed using *Antechamber* to generate suitable topologies.^38^ The complete system topologies, for both protein–ligand complexes and unbound protein, were then generated within the LEaP module of AmberTools24, applying the protein.ff19SB force field and general AMBER force field (GAFF) for the ligands.^39,40^ Missing hydrogen and other atoms were also added during this preparation. Systems were neutralized with Na^+^ counterions and solvated in an octahedral box of Transferable Intermolecular Interaction Potential 3 Points (TIP3P) model water molecules, with a minimal wall distance of 12 Å. Temperature (310.15 K) and pressure (1 atm) were maintained using the Berendsen barostat and thermostat.^41^ Covalent bonds involving hydrogen atoms were constrained using the SHAKE algorithm, enabling a 2 fs time step to integrate Newton’s equations, as recommended by the Amber package.

MD calculations were performed using a GPU-accelerated AMBER engine (pmemd.cuda), on an Ubuntu 22.04.5 Workstation with an Nvidia GeForce RTX 4090 GPU, achieving 325 ns/day.^42^ The simulation protocol began with initial structure optimization, followed by a sequential 50 ps heating step at 310.15 K, 50 ps constant volume equilibration, and 500 ps constant equilibration at 1 atm. Subsequently, several independent 100 ns MD simulations were performed for all complexes. Triplicate 100 ns MD simulations were performed for DPP-4 complexes with the three selected candidates, reference drug (alogliptin), and the negative control (acetaminophen) using the same computational protocol described above. Frames were saved at 100 ps intervals for subsequent analysis.

#### 2.4.3 Trajectory Analysis

Average C, C-α, and N Root-Mean-Square Deviation (RMSD) and Root-Mean-Square Fluctuation (RMSF) values were calculated from simulations using the CPPTRAJ module in AmberTools24 and subsequently plotted using OriginLab version 9.0 (OriginLab Corporation, Northampton, MA, USA). To comprehensively assess the flexibility of DPP-4 when complexed with the compounds, the MDLovoFit package was employed (Institute of Chemistry, University of Campinas).^43^ VMD^44^ and PyMOL were used for visualization and to generate MD images.

To compare total protein–ligand binding affinities, the absolute Gibbs binding free energy (Δ*G*_bind_) and the sum of energies of the complex were calculated using the molecular mechanics with Generalized Born and Surface-Area (MM-GBSA) solvent method. This technique was also used to estimate the individual energy contribution of residues toward the overall energy.^45,46^ MM-GBSA calculations utilized the last 50 ns of the trajectory, corresponding to 100 frames. All reported values represent mean data from three independent runs ± standard deviation.

### 2.5 Passage of Compounds across the Lipid Bilayer

#### 2.5.1 Simulation of Free Energy Transfer Profile by Umbrella Sampling

Permeability of the compounds across a biological membrane was assessed by calculating the permeation free energy (PFE) using the Umbrella Sampling method within the AMBER software package.^47^ A lipid bilayer model mimicking an enterocyte membrane was constructed based on the reported molar ratios.^48–50^ For this purpose, 2-dioleoyl-*sn*-glycero-3-phosphocholine (DOPC), dioleoylphosphatidylethanolamine (DOPE), palmitoylsphingomyelin (PSM), and 1,2-dioleoyl-*sn*-glycero-3-phospho-l-serine (DOPS) were assembled in a 5:3:1:1 molar ratio using PACKMOL-Memgen.^51^ Initial system setup involved positioning each parametrized ligand at the bilayer lipidic center (*z* ∼ 0 Å), generating its topology and coordinate files. The system was simulated using TIP3PBOX water (5 Å radius) and neutralized by adding counterions. The lipids were modeled using the Lipid21 force field.^52^ Following energy minimization, systems underwent a progressive heating phase (0 to 100 K for 5 ps, and then, up to 310.15 K for 100 ps), concluding with a 100 ps constant volume equilibration. Steered molecular dynamics were then performed to move each ligand along the *z*-axis of the bilayer from its initial position (*z* = 0 Å) to a final position (*z* = 35 Å), by applying a stepwise external force to its center of mass. From these trajectories, 35 initial configurations (windows) were extracted along the reaction coordinate, with approximately 1 Å separation between the centers of adjacent windows.

Subsequently, for each window, equilibrium MD was performed for 2 ns at 310.15 K, with harmonic restraints using a force constant of 2.5 kcal/mol/Å^2^. Temperature control was achieved using the Langevin algorithm, while pressure control was performed using the Berendsen barostat Monte Carlo. Periodic boundary conditions and the particle mesh Ewald (PME) method were employed for long-range electrostatic interactions, with a cutoff distance of 10.0 Å. The SHAKE algorithm was used to constrain bonds involving hydrogen atoms. After completing all MD windows, the PMF (kcal/mol) was reconstructed from these distributions using the Weighted Histogram Analysis Method (WHAM).

#### 2.5.2 Simulation of Compounds Binding to a Membrane

Long-time MD simulations were performed to investigate the spontaneous association and equilibrium behavior of the compounds with a model enterocyte bilayer. This lipid bilayer was also constructed using PACKMOL-Memgen with a DOPC:DOPE:PSM:DOPS molar ratio of 5:3:1:1. The solvated system, using the TIP3PBOX water model, included 15 ligand molecules positioned without restrictions in the aqueous phase. System preparation involved a rigorous step-by-step minimization and equilibration protocol to ensure stability. This began with two solvent minimization phases with harmonic restraints of 25 kcal/mol Å^2^ and 5 kcal/mol Å^2^, respectively, to gradually relax the water molecules and remove steric hindrance. Subsequently, a two-stage minimization was performed with all components (ligand, lipids, water, and ions), applying 5 kcal/mol harmonic restraints on the ligand and bilayer. Equilibration followed, starting with a 5 ps NVT (constant number of atoms, volume, and temperature) phase with gradual heating from 0 to 100 K, followed by another NVT phase up to 310.15 K. Finally, a 4.88 ns NPT (constant number of atoms, pressure, and temperature) equilibration at 310.15 K allowed all components to move freely. Each system then underwent a 200 ns production MD, maintaining these conditions. Trajectories from these MD were analyzed using a custom-developed Python script. We calculated several parameters to characterize ligand permeability using a 4 Å cutoff distance. These include the total number of contacts between the ligands and membrane atoms per frame, the number of contacts per leaflet, and the ligand insertion depth relative to the bilayer center (*z*-axis) to provide a dynamic overview of ligand interactions and permeation events.

## 3 Results and Discussion

### 3.1 Virtual Screening of Compounds against DPP-4

#### 3.1.1 Database Selection and Ligand Preparation

The efficacy of virtual screening hinges on the appropriate selection of ligand sets, which directly impacts the relevance of “*in silico*” generated data. For this reason, we selected the PubChem database as a resource for identifying potential novel DPP-4*i* candidates. This platform has an extensive curated library of compounds (122 million as of August 1, 2025) and comprehensive compound information, including chemical and physical properties, bioactivity, pharmacology, and toxicology data. Its integration with relevant external databases made it an excellent resource for this antidiabetic drug discovery effort.

We filtered the PubChem database using the keyword “*natural*” to identify 836,156 compounds derived from natural sources. This focus enhances translational prospects, as these scaffolds have historically served as platforms for successful drug development and often exhibit favorable safety profiles. The natural chemical diversity could also address the safety limitations of current synthetic DPP-4*i*. Subsequently, the data set was filtered in a multistep process as illustrated in Scheme 1. First, 304,416 commercially available compounds were selected. Next, we extracted 30,699 compounds classified by biological activity across a broad spectrum of therapeutic targets. Of these, 3,888 compounds exhibited nanomolar activity, and 26,811 had micromolar activity. This strategic selection enriched our screening library with pharmaceutically validated compounds, representing a critical methodological advantage. By combining GPU acceleration with intelligent prefiltering based on proven therapeutic potential, this approach optimizes computational efficiency while minimizing false positives, ensuring that computational resources focus on molecules with the highest probability of experimental success.

**Scheme 1:**
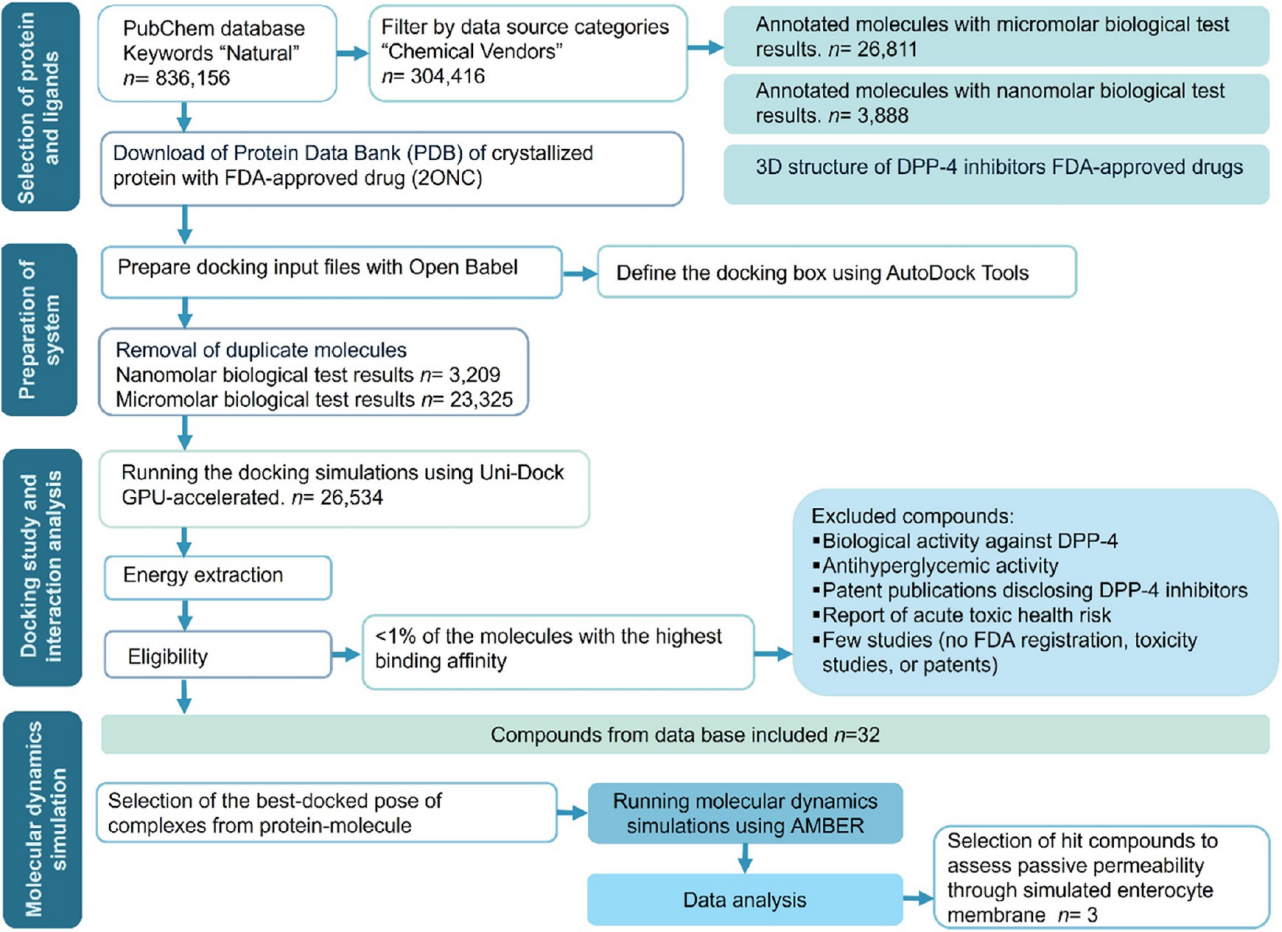
Designed Workflow for the Virtual Screening and Molecular Dynamics Simulations of Inhibitor Candidates for DPP-4

#### 3.1.2 Docking Analysis

Following compound preparation using Open Babel software,^36^ duplicate molecules were removed from the databases, resulting in a final library of 23,325 μM compounds and 3209 nM compounds. Exhaustive virtual screening on all 26,534 ligands was performed using the GPU-accelerated Uni-Dock.^30^ Docking of these compound libraries was performed simultaneously with FDA-approved gliptins serving as reference parameters for comparison. The distribution of docking energy scores (kcal/mol) and frequencies for both libraries is presented in Figure 2.

**Figure 2:**
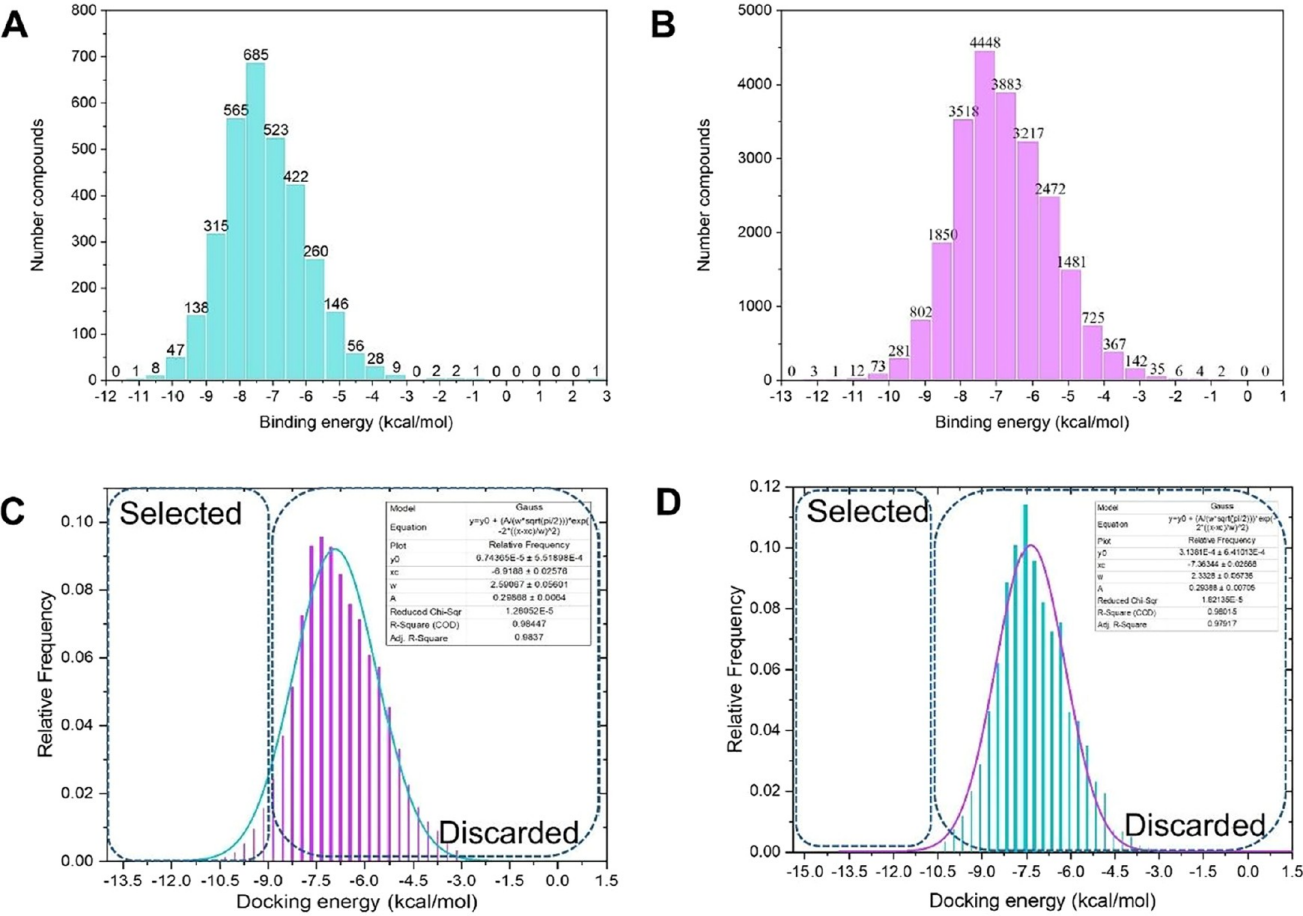
Uni-Dock virtual screening results for the DPP-4*i*. (A) Present the docking score histograms (kcal/mol) for 23,325 compounds with reported micromolar bioactivity. (B) 3209 Compounds with nanomolar bioactivity, respectively. (C and D) Illustrate the frequency distribution graphs of these docked compounds against DPP-4 across the range of docking scores for the micromolar and nanomolar libraries, respectively.

As we expected for a large-scale virtual screening, docking scores across both databases were broadly distributed (Figure 2). Nanomolar compounds exhibited binding energies ranging from −11.22 to 2.50 kcal/mol, while the micromolar compound set showed energies ranging from −12.08 to 52.54 kcal/mol. FDA-approved DPP-4*i* displayed binding energies of −9.2 to −6.4 kcal/mol, consistent with previous reports.^53^ Among screened compounds, 582 compounds had a docking score higher than the reference inhibitors, and an additional 16,546 compounds showed comparable binding energies. This initial assessment suggests the presence of numerous potential high-affinity DPP-4 binders within our natural compound libraries, validating that our integrated computational workflow is an effective strategy for identifying novel DPP-4*i* with desirable binding profiles. Extension of this computational framework to synthetic libraries in subsequent studies could provide a comprehensive assessment of the chemical space available and complement our natural product-focused approach.

Among all screened compounds, CID 135524769 demonstrated the highest binding affinity to DPP-4 (−12.08 kcal/mol), followed by CID 158365 (−11.84 kcal/mol), CID 60775 (−11.83 kcal/mol), CID 70186, and CID 42611190 (−11.78 kcal/mol) (Supporting Table S1).

In accordance with Scheme 1, stringent exclusion criteria were applied to refine the pool. This process removed candidates with existing patents for antihyperglycemic or DPP-4 inhibitory activity, evidence of acute toxicity, unavailable toxicology data, or nonpatented compounds. The filtering process reduced the initial 189 candidates (Supporting Table S1) to a final data set of 32 compounds for subsequent detailed analysis (Supporting Table S2). All selected compounds are registered but not subject to US FDA approval (“off-label”).

To elucidate the intermolecular interactions critical for DPP-4 inhibition, we analyzed the binding modes of three natural repurposed compounds: OSU-03012 (CID 10027278), EPZ005687 (CID 60160561), and bemcentinib (CID 46215462), initially identified for their antitumor and antimicrobial activities.^54–56^ These candidates exhibited superior docking scores (−9.97, −11.12, and −10.16 kcal/mol, respectively) compared to reference drugs tested. Detailed binding analysis was focused on alogliptin (CID: 11450633, CAS: 850649–61–5) (Figure 3), a well-characterized DPP-4*i* known for its specific binding to S1′, S2′, S1, and S2 subsites of the enzyme, and high inhibitory activity (IC_50_ < 10 nM).^57,58^

**Figure 3:**
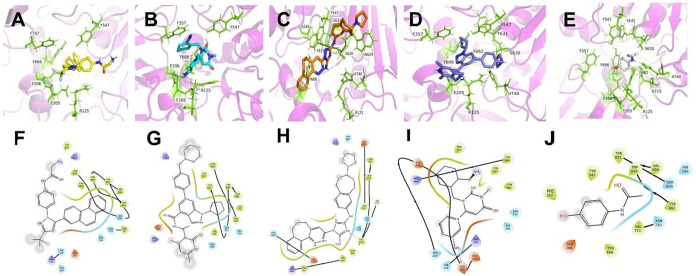
Molecular docking interactions between DPP-4 and lead compounds compared with reference inhibitors. This figure illustrates the binding modes and molecular interactions of DPP-4 with three candidate compounds and two reference molecules through complementary 3D and 2D visualization approaches. Panels (A–E) (Top row): (A) CID 10027278, (B) CID 60160561, (C) CID 46215462, (D) Alogliptin (reference DPP-4 inhibitor), and (E) Acetaminophen (negative control) bound to DPP-4. Panels (F–J) (Bottom row): 2D ligand interaction diagrams created with Maestro Visualizer v14.3, displaying the same compounds in corresponding order (F through J).

Analysis of the top candidate binding modes (Figure 3) revealed both common and distinct interaction patterns within the DPP-4 active site. All three compounds engaged hydrophobic residues lining the substrate binding pocket (Tyr631, Val656, Trp659, Tyr662, and Val711), and extended contact to Tyr547 (S1′ subsite) and Trp629 (S2′ subsite). The negatively charged residues, Glu205 and Glu206, critical for N-terminal substrate recognition,^59^ were consistently involved in binding across all candidates (Figure 3). Beyond these commonalities, specific interactions with other regions of the binding pocket varied among the three compounds. CID 10027278 oriented toward the polar side chains of the catalytic triad residues (His740, Asn710, and Ser630; Figure 3A,3F); CID 60160561 formed polar interactions with Ser630 (Figure 3B,G); while CID 46215462 established a hydrogen bond via the N3 of the triazole ring with Tyr631 (S1 subsite), and had additional contact with His740 (Figure 3C,3H).

Compared to alogliptin, candidate compounds demonstrated more extensive interactions within the DPP-4 active site. While alogliptin formed a hydrogen bond with Glu205 via its aminopiperidine ring, it showed limited proximity to other active site residues within 4 Å, and fewer bidirectional contacts (Figure 3D,3I). These suggest our top candidates may engage more comprehensively with the binding pocket. Furthermore, acetaminophen confirmed its utility as a negative control. Despite a relatively favorable docking score (−5.71 kcal/mol), its interactions were restricted to Asn710 and Trp659 (Figure 3E,J), demonstrating a limited binding profile for nonspecific compounds.

Overall, docking analysis confirms the effectiveness of our curation strategy, with candidate compounds demonstrating strong binding affinity and appropriate DPP-4 active site localization, revealing binding modes comparable to current therapeutic inhibitors used in T2DM.

### 3.2 Molecular Dynamics Analysis

To investigate the stability and dynamic interactions of selected compound candidates within the DPP-4 active site, we performed 100 ns MD on the 32 best protein–ligand complexes. FDA-approved reference controls and acetaminophen were also included. The following sections detail the findings obtained from the trajectory analysis.

#### 3.2.1 Binding Free Energy

##### 3.2.1.1 Intermolecular Interaction Energies

To estimate the affinity between DPP-4 and potential inhibitors, we calculated the Δ*G*_bind_ using the MM-GBSA method.^43^ The Δ*G*_bind_ values for screened compounds ranged from −58.18 to −5.78 kcal/mol (Supporting Table S2). Eight compounds exhibited higher binding affinity (−58.18 to −39.66 kcal/mol) compared to benchmark FDA-approved drugs (−24.83 to −38.4 kcal/mol) (Figure 4). Furthermore, 15 candidate complexes fell within the binding energy range observed for the reference drugs. As expected, the negative control, acetaminophen, showed significantly weaker binding (−8.50 kcal/mol), validating the accuracy and sensitivity.

**Figure 4:**
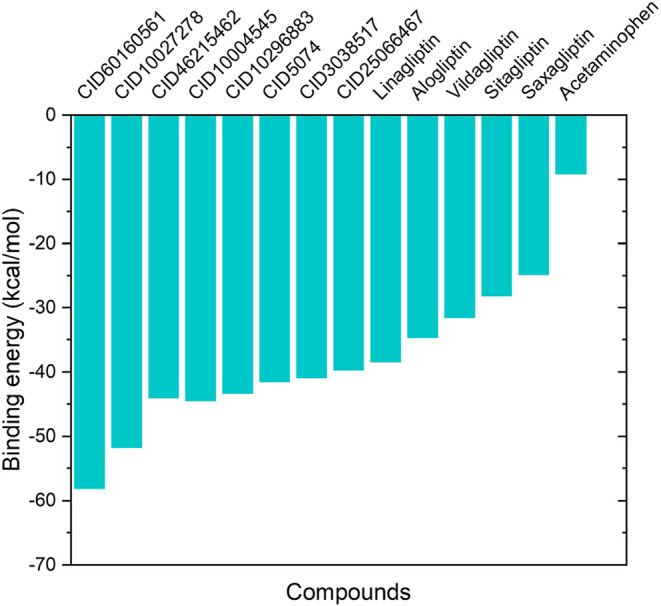
MM-GBSA binding free energies of DPP-4 inhibitors. Comparison of calculated binding free energies (Δ*G*_bind_, kcal/mol) for FDA-approved inhibitors (CID identifiers) and natural compound candidates. More negative values indicate stronger binding affinity to the DPP-4 enzyme. Acetaminophen was included as a negative control.

CID 60160561 exhibited the most favorable binding free energy (Δ*G*_bind_ of −58.18 ± 3.47 kcal/mol), followed by CID 10027278 (Δ*G*_bind_: −51.73 ± 5.379 kcal/mol) and CID 46215462 (Δ*G*_bind_: −44.05 ± 6.57 kcal/mol). Given their superior binding potential, the detailed discussion will focus on these three compounds, with alogliptin and acetaminophen included for comparative analysis and validation throughout the subsequent sections.

#### 3.2.2 Structural Stability and Flexibility of Complexes

##### 3.2.2.1 RMSD Analysis

To assess the stability of protein–ligand complexes relative to their initial structural conformation over 100 ns trajectories, we calculated the RMSD for the protein backbone. The RMSD of each complex (magenta) was directly compared to that of the unbound (free) DPP-4 (blue). Free DPP-4 stabilized around 40 ns and maintained an average RMSD of 2.74 ± 0.11 Å during the equilibration (Figure 5 and Supporting Movie 1). Remarkably, the candidate compounds exhibited superior structural stabilization compared to both free DPP-4 and the complex with alogliptin once equilibrium was reached. DPP-4 in complex with CID 10027278 (Figure 5A) and CID 60160561 (Figure 5C) reached a conformation comparable to that of the free protein around 60 ns (2.51 ± 0.10 Å) and 40 ns (2.31 ± 0.17 Å), respectively. The DPP4-CID 46215462 complex equilibrated rapidly (∼20 ns) and maintained the lowest RMSD values (2.13 ± 0.09 Å), suggesting high ligand-induced stability within the active site (Figure 5E).

**Figure 5:**
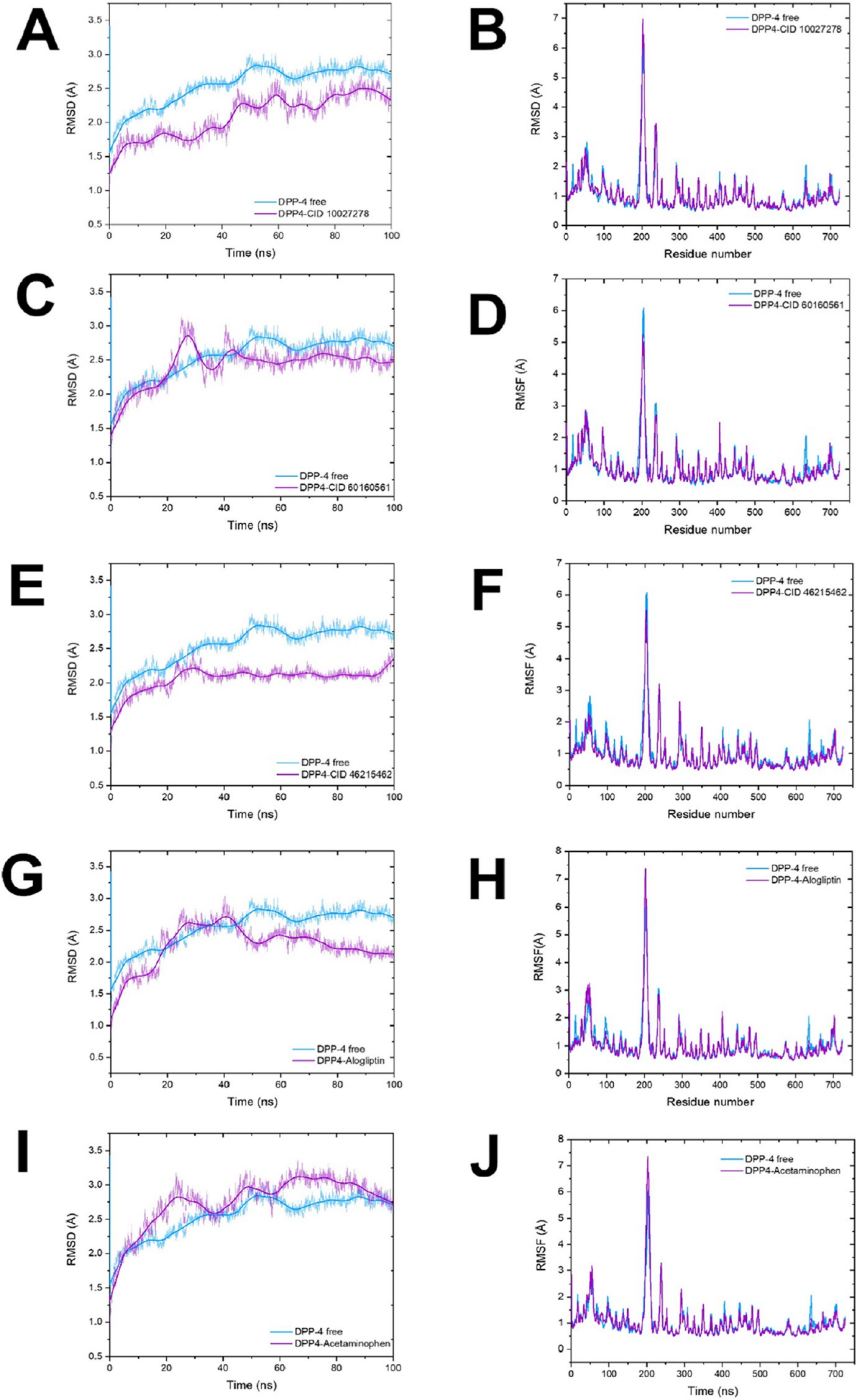
Stability and flexibility of DPP-4 and its complexes during 100 ns MD. This figure shows average root-mean-square deviation (RMSD) and root-mean-square fluctuation (RMSF) for the backbone (C, Cα, N, O) atoms of dipeptidyl peptidase-4 (DPP-4) across the three molecular dynamics simulation runs. Free DPP-4 is shown in blue, and its complexes are shown in magenta. Panels A, C, E, G, and I show RMSD as a function of simulation time for DPP-4 free and in complexes with (A) CID 10027278, (C) CID 60160561, (E) CID 46215462, (G) alogliptin, and (I) acetaminophen. Figures B, D, F, H, J display RMSF as function of residue number for DPP-4 free and in complexes with (B) CID 10027278, (D) CID 60160561, (F) CID 46215462, (H) alogliptin, and (J) acetaminophen small insects within the RMSF plots highlight regions with significant fluctuations, corresponding to residue ranges of approximately 192–211 and 635–705.

In contrast, the RMSD of the DPP4-alogliptin complex exhibited fluctuations during the initial phase but eventually stabilized after 50 ns, with average values of 2.27 ± 0.12 Å (Figure 5G). As anticipated, the DPP4-acetaminophen system was the only compound that exhibited higher RMSD values, ranging from 0.76 to 3.36 Å (Figure 5I). The escalating correlates with the observed dissociation of acetaminophen from the active site (Supporting Movies S2), confirming its lack of specific binding affinity. The dissociation of ligand was absent in complexes with high-affinity ligands like CID 10027278 (Supporting Movies S3). Collectively, RMSD analysis demonstrates that 100 ns simulations were sufficient to achieve structural stability for both the free protein and all protein–ligand complexes, providing a robust foundation for subsequent analyses. Importantly, the conformational stabilization of the protein structure induced by the lead candidate suggests enhanced protein–ligand interactions.

##### 3.2.2.2 RMSF and MDLovoFit Analysis

Beyond overall structural stability, we analyzed the local flexibility of protein residues by calculating the RMSF of backbone atoms, comparing protein–ligand complexes (magenta) to free DPP-4 (blue) (Figure 5). Two regions exhibited high intrinsic flexibility in free DPP-4: amino acids 192 to 211, particularly Glu205 and Glu206 in the S2 subsite (with RMSF values of ∼8 Å), and residues near the C-terminal loop of the catalytic α/β-hydrolase domain (635–703). The proximity of the 192–211 loop to the active site enables it to function as an extended arm, blocking substrate entry. This region contains a highly conserved motif (Asp200, Trp201, Val202, Tyr203, Glu204, Glu205, and Glu206), and point mutations at Glu205 and Glu206 are known to abolish enzymatic activity.^53^ MDLovoFit analysis^43^ confirmed pronounced mobility in the 192–211 region, identifying it among the 30% most mobile atoms throughout the simulation (Figure 6).

**Figure 6:**
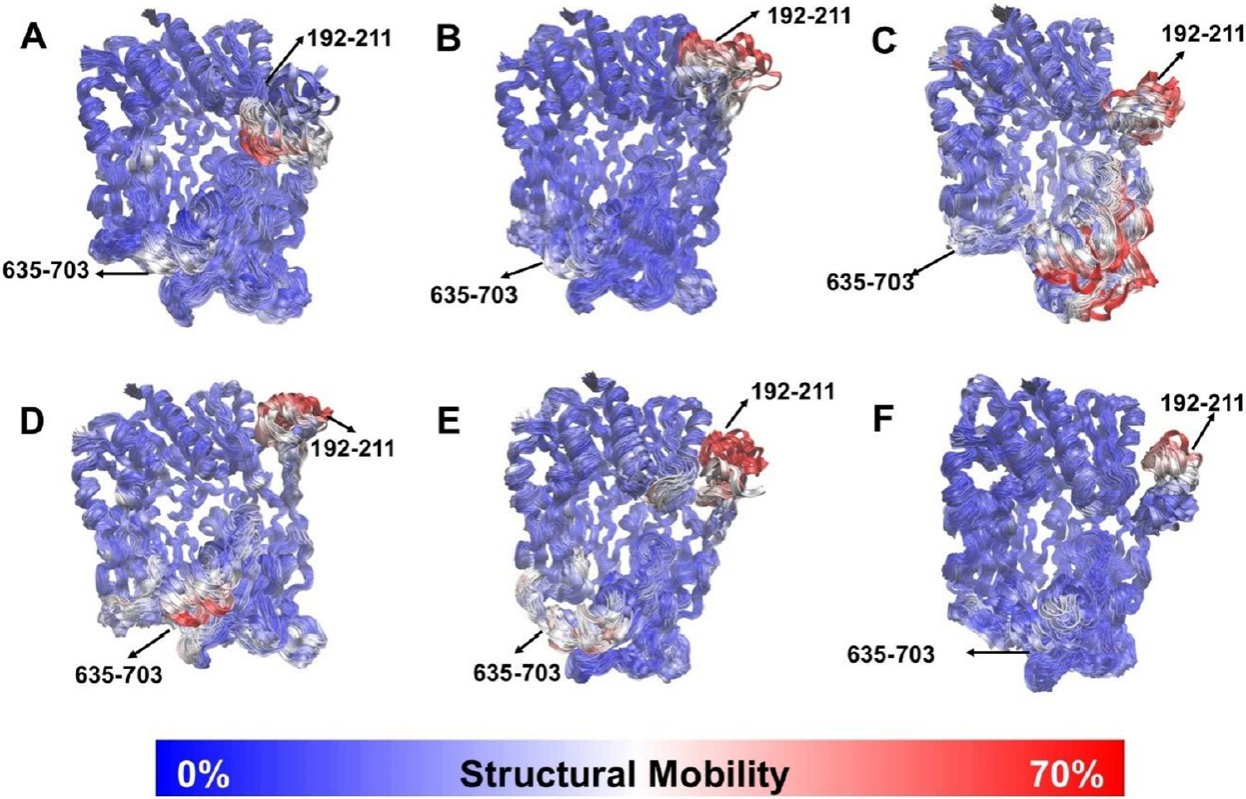
Dynamic flexibility of dipeptidyl peptidase-4 upon ligand binding. 3D visualizations show the flexibility of DPP-4 residues: (A) free, and in complex with (B) CID 10027278, (C) CID 60160561, (D) CID 46215462, (E) alogliptin, and (F) acetaminophen. Residues are colored based on their Root-Mean-Square Fluctuation (RMSF) relative to the initial structure after alignment, with the 70% least mobile atoms in blue and the 30% most mobile atoms in red. Visualizations were generated using MDLovoFit and VMD outputs.

Average backbone RMSF showed no significant difference in mobility of the C-terminal loop residues (635–703) between free DPP-4 and complexes with test compounds (Figure 5B, 5D, 5F, and 5H). However, MDLovoFit analysis revealed distinct structural deviations in this region depending on the ligands. While DPP-4 core maintained a preserved conformation across simulations (70% least displaced atoms in blue) free DPP-4­(Figure 6A), and complexes with CID 10027278 (Figure 6B) or acetaminophen (Figure 6F) showed lower C-terminal mobility compared to complexes with CID 60160561 (Figure 6C), CID 46215462 (Figure 6D), and alogliptin (Figure 6E). Conformational changes in the C-terminal region of some complexes suggest potential catalytic misalignment, warranting further investigation.

#### 3.2.3 Energetic Components and Free Energy Decomposition

To elucidate the nature of the interactions, we performed binding free energy decomposition analysis, calculating the individual contributions of the electrostatic (Δ*E*_ele_), van der Waals (Δ*E*_vdw_), polar solvation (Δ*G*_pol_), and nonpolar solvation (Δ*G*_nonpolar_) energies to the total ΔG_bind_, alongside the energetic contributions of residues. Table 1 presents the subtotal energy (Δ*G*_subtotal_) data for the top-scoring candidates compared with alogliptin and acetaminophen.

**Table 1:** Free Energy Decomposition for the DPP4-Compound Complex in Terms of the Contributions from the Electrostatic Interaction Energy (Δ*E*_ele_), van der Waals energy (Δ*E*_vdw_), the Polar Solvation Free Energy (Δ*G*_polar_), and the Non-Polar Solvation Free Energy (Δ*G*_non‑polar_)­^a^

Compound	*ΔE*_ele_	*ΔE*_vdW_	*ΔG*_pol_	*ΔG*_nonpolar_	*ΔG*_bind_
CID 60160561	–68.04 ± 9.38	–67.71 ± 3.34	83.72 ± 7.93	–6.15 ± 0.18	–58.18 ± 3.47
CID 10027278	–321.4 ± 26.28	–54.82 ± 3.15	329.81 ± 23.39	–5.23 ± 0.29	–51.73 ± 5.38
CID 46215462	–28.65 ± 15.61	–53.76 ± 6.80	43.00 ± 14.26	–4.64 ± 0.49	–44.05 ± 6.57
Alogliptin	–66.70 ± 13.22	–36.66 ± 4.31	72.63 ± 10.80	–9.91 ± 0.39	–28.76 ± 3.15
Acetaminophen	74.79 ± 35.54	–11.47 ± 5.09	–70.54 ± 35.16	–1.65 ± 0.76	–9.15 ± 4.90

a Energies are in kcal/mol.

Δ*G*_bind_ for most ligands is driven primarily by favorable nonpolar terms (Δ*E*_vdw_ + Δ*G*_nonpolar_). While direct electrostatic interactions are often favorable, they are counteracted by unfavorable polar solvation energies, which represent desolvation penalties. This indicates that desolvation costs modulate the net polar contribution to binding affinity. In contrast, acetaminophen exhibits a different profile characterized by unfavorable electrostatic interactions (74.79 ± 35.54 kcal/mol) compensated by favorable polar solvation (−70.54 ± 35.16 kcal/mol), resulting in a weaker overall binding.

##### 3.2.3.1 Alogliptin Analysis

For alogliptin, the total binding free energy was dominated by favorable nonpolar terms, which contributed −40.59 ± 4.71 kcal/mol. Net polar contribution remained unfavorable (4.84 kcal/mol), consistent with the general desolvation penalties counteracting electrostatic attractions observed across most ligands (Table 1). Per-residue analysis (Figure 7A) revealed Ser209 (S2 extensive subsite) and Glu206 (S2 subsite) as fundamental binding, with significant electrostatic interactions from Glu206 (−15.04 kcal/mol) and Glu205 (−6.88 kcal/mol). Favorable van der Waals contributions were provided by Ser209, Phe357, and Arg358 (−2.45, −2.54, and −2.68 kcal/mol, respectively) (Supporting Figure S1A). These interactions across S1, S2, S1′, and S2′ subsites, particularly with Glu205 and Glu206, are consistent with previous reports on maximizing interaction strength.^60^

**Figure 7:**
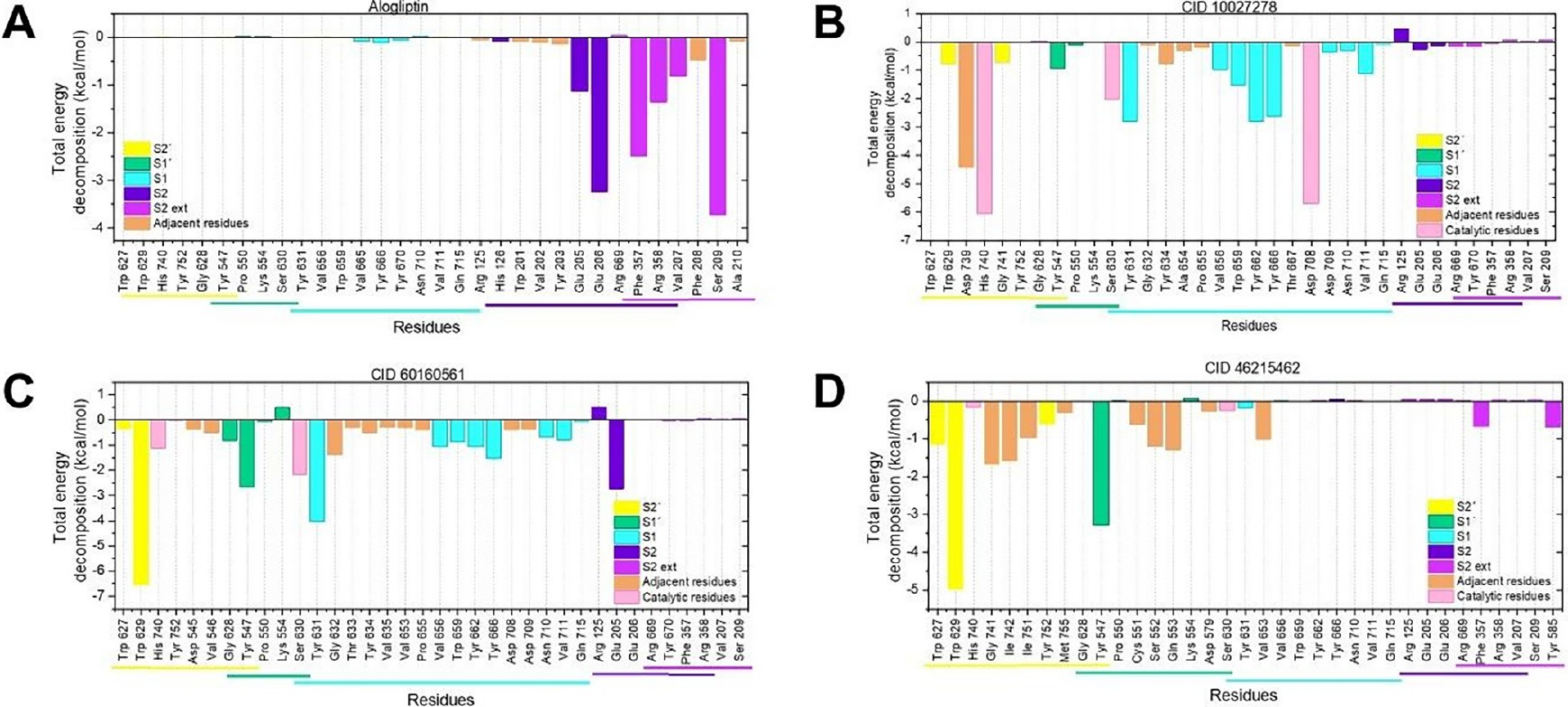
Per-residue binding free energy decomposition analysis of DPP-4 inhibitor interactions. Bar plots showing the contribution of individual DPP-4 active site residues to the total binding free energy (Δ*G*_bind_) for: (A) alogliptin, (B) CID 10027278, (C) CID 60160561, and (D) CID 46215462. Residues are color-coded according to their assigned DPP-4 subsite (S1, S2, S1′, S2′, and S2 ext, as indicated in the legend), with colored horizontal bars beneath the *x*-axis delineating residues belonging to each subsite. Negative values indicate favorable contributions to binding. Energy decomposition was calculated using the Molecular Mechanics-Generalized Born Surface Area (MM-GBSA) method.

##### 3.2.3.2 CID 10027278 Analysis

The binding profile of CID 10027278 also showed predominant nonpolar contributions (−60.06 kcal/mol) (Table 1). Per-residue decomposition shows catalytic triad residues His740 (Δ*E*_vdw_: −44.56 kcal/mol) and Asp708 (Δ*E*_ele_: −44.56 kcal/mol) as major contributors (Figure 7B and Supporting Figure S1B). Favorable van der Waals interactions from hydrophobic S1 subsite residues (Ser630, Tyr631, Val656, Trp659, Tyr662, Tyr665, Tyr666, Asn710, and Asn715) complemented the strong nonpolar component. Notably, Asp739, adjacent to the DPP-4 pocket, provides a favorable net energy contribution (Δ*E*_subtotal_: −4.42 kcal/mol), through electrostatic interaction (Supporting Figure S1B). The strong binding to key charged residues near the active site aligns with reports showing such interactions enhance both binding rate and stability.^60,61^ These dominant nonpolar contributions and optimal positioning suggest CID 10027278 could exhibit competitive DPP-4 inhibition.

##### 3.2.3.3 CID 60160561 Analysis

In turn, CID 60160561 binding was similarly driven by highly favorable net nonpolar terms, which collectively contributed a value of −73.87 kcal/mol (Table 1). Per-residue energy decomposition (Figure 7C and Supporting Figure S1C) revealed that Trp629 contributed a favorable subtotal binding energy (−4.96 kcal/mol), primarily from favorable van der Waals dispersion interaction (−5.17 kcal/mol). Additional stability was provided by side chains of Tyr631 and Ser630 (S1 subsite), Glu205 (S2 subsite), and Tyr547 (S1′ subsite). The interaction with both S2′ and S1′ subsites residues is particularly relevant, as extended interactions in these regions have been suggested to increase the inhibition potency potentially.^56,62^

##### 3.2.3.4 CID 46215462 Analysis

CID 46215462 binding was primarily attributed to favorable net nonpolar terms totaling −58.40 kcal/mol. The most significant interactions occurred through Trp629 (S2′ subsite) and Tyr547 (S1 subsite) (Figure 7D), both exhibiting a favorable Δ*E*_subtotal_ from van der Waals interactions (Supporting Figure S1D). Given that Tyr547 facilitates tetrahedral oxyanion intermediate stabilization during catalysis,^63^ interaction with this residue could potentially impact the catalytic efficiency of the enzyme.

It is worth noting that residues adjacent to the DPP-4 pocket (Gly741, Ile742, Gly549, Cys551, and Gln553) also contributed to complex stabilization through favorable van der Waals interactions (Figure 7D and Supporting Figure S1D). Although these may enhance ligand binding, the precise mechanism of inhibition for CID 46215462 requires *in vitro* confirmation. Establishing an inhibition mechanism distinct from competitive binding would be particularly relevant, given its unusual nature among characterized DPP-4 inhibitors.^64^

In summary, the detailed decomposition energy analysis, recognized for predicting biological potency,^65^ strongly supports the premise of the potential of CID 10027278, CID 60160561, and CID 46215462 as effective DPP-4*i*. Their distinct binding mechanisms, dominated by van der Waals forces and nonpolar interactions, along with active site engagement, position them as promising candidates for development as novel antidiabetic agents.

#### 3.2.4 Analysis of Interaction Persistence

High-affinity binding is often related to the ability of a ligand to stay bound to a protein for extended times, resulting in stable interactions that resist dissociation.^66^ When these prolonged interactions involve specific amino acids within the active site, the ligand can disrupt catalytic activity and, consequently, elicit a more sustained biological effect.^62,67^ To evaluate this crucial aspect of binding and stability, we investigated the fraction of time each ligand-residue interaction was maintained throughout the 100 ns MD trajectory.

##### 3.2.4.1 Alogliptin

As illustrated in Figure 8A, alogliptin established persistent interactions with Ser209, Glu206, Phe357, Arg358, Glu205, Val207, and Phe208 throughout the 100 ns simulations, confirming the significant energetic contribution observed in prior per-residue decomposition analysis. Notably, high contact frequency does not always correlate with a proportional high energetic contribution. These can occur due to weak but persistent conventional interactions or unfavorable desolvation penalties. While MM-GBSA quantifies classical electrostatic and van der Waals interactions, “unconventional” forces, such as CH-π interactions or halogen bonds, can provide substantial cumulative stability despite not being explicitly quantified.^68^ In line with this, alogliptin maintained interaction with Arg669 (S2 extensive subsite) and His126 (adjacent to S2 subsite) for over 95% of simulation time, despite minimal individual energetic contributions. This suggests that their collective, persistent, though individually minor, contributions are crucial for overall stabilization and prolonged residence time.

**Figure 8:**
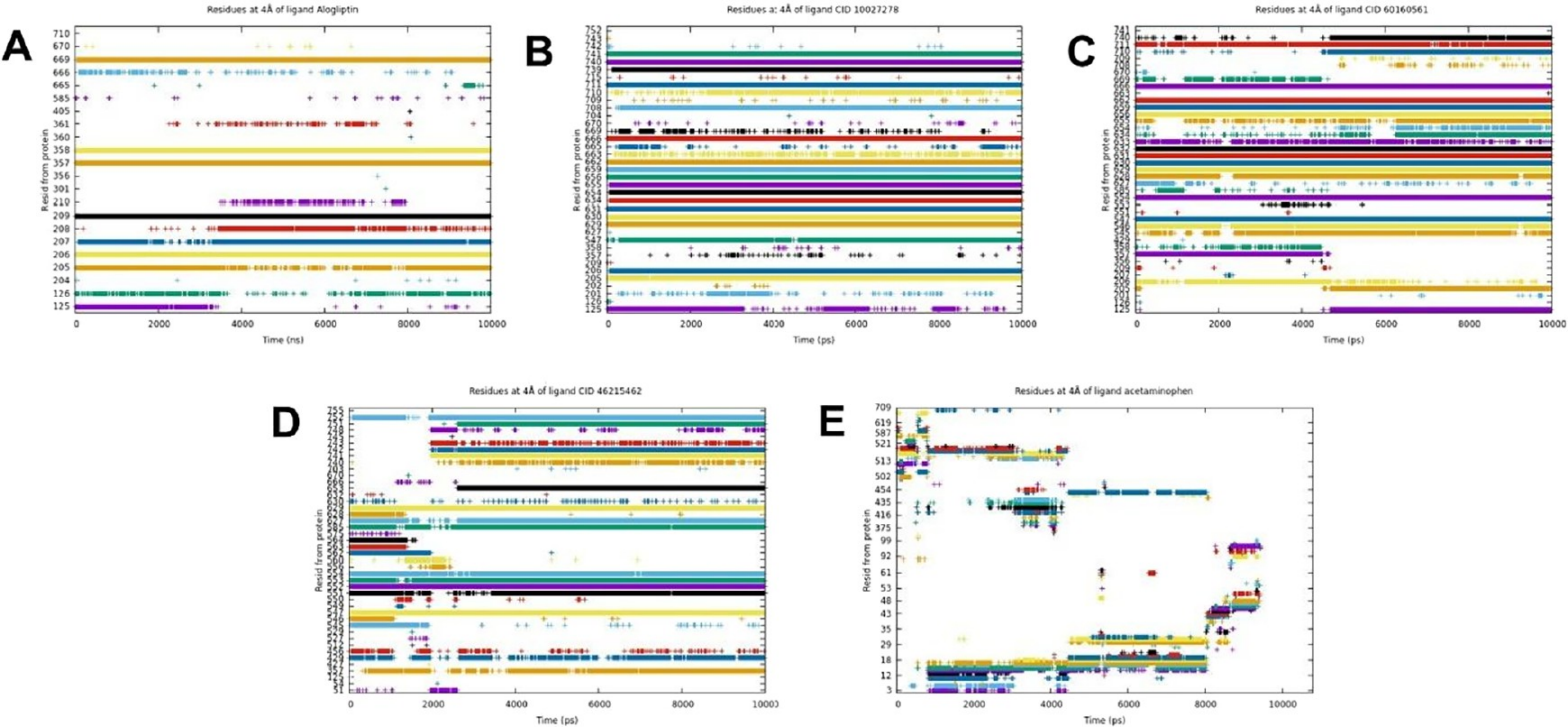
Ligand-residue contact matrix from molecular dynamics simulations. This figure illustrates the persistent interactions profile between individual DPP4-residues and (A) alogliptin, (B) CID 10027278, (C) CID 60160561, (D) CID 46215462, and (E) acetaminophen with DPP-4 residues over 100 ns of MD trajectory. Each horizontal bar indicates the frames during which a specific DPP-4 residue-maintained contact with the ligand within a 4 Å distance. Longer or more prominent bars signify higher frequency and duration of contact, highlighting stable, persistent interactions between the ligand and that residue throughout the simulation.

##### 3.2.4.2 Compounds Candidate for DPP-4 Inhibitors

CID 10027278, CID 60160561, and CID 46215462 displayed consistent and persistent binding with key DPP-4 residues (Figure 8B–8D), confirming the per-residue energy decomposition results. Major contributors to Δ*E*_bind_ and additional interaction residues maintained contact for 80–100% of the 100 ns simulation. The contact matrix analysis revealed consistent interactions, even with residues yielding limited net energetic contributions, extending beyond primary binding sites.

##### 3.2.4.3 CID 10027278

CID 10027278 displayed an additional persistent interaction with Arg125 (S2 subsite) for 100% of the trajectory (Figure 8B). The extensive and multifaceted interaction profile across subsites S1 (Ser630, Tyr631, Val656, Trp659, Tyr662, Tyr666, Asn710, Asn711, and Gln715), S2 (Arg125, Glu205, and Glu206), S1′ (Tyr547 and Pro550), and S2′ (His740 and Trp629), and S2 extensive (Arg669) suggest potent and selective inhibitory effects. This broad binding pattern resembles high-potency inhibitors like linagliptin, which shows 8-fold higher activity than alogliptin and 10,000-fold DPP-4 selectivity due to extensive interactions with the S2 subsite, a region largely absent in similar peptidases.^69,70^ These characteristics position CID 10027278 as a promising candidate for *in vitro* studies and therapeutic development.

##### 3.2.4.4 CID 60160561

CID 60160561 also established a broad range of persistent contacts throughout the simulation (Figure 8C), confirming energy decomposition findings. Beyond previously highlighted residues, Arg125 and Glu206 (S2 subsite) maintained contact for approximately 50 and 70 ns, respectively, while Lys554 (S1′ subsite) interacted for the entire simulation. Engagement extended across the hydrophobic S1 pocket (Ser630, Tyr631, Val656, Trp659, Tyr662, Tyr666, Asn710, and Asn711), S2 (Arg125, Glu205, and Glu206), S1′ (Gly628, Tyr547, and Lys554), and S2′ (Trp629) subsites, positioning CID 60160561 as another promising antidiabetic candidate for targeting DPP-4.

##### 3.2.4.5 CID 46215462

The interaction matrix revealed interactions of CID 46215462 binding both inside and outside the active site (Figure 8D). Specifically, Arg429, located outside the canonical active site, exhibited remarkably persistent binding for at least 80% of the trajectory, sustained by favorable hydrophobic forces. Tyr666 (S1 subsite) and Lys554 (S1′ subsite) maintained interactions throughout the entire simulation, alongside pocket-adjacent residues (Cys551, Ser552, Gln553, Gly741, Ile742, and Ile743). Binding extended to subsites S1 (Ser630 and Tyr631), S1′ (Tyr547 and Lys554), S2′ (His740, Trp627, Trp629, and Tyr752), and extended S2 (Phe357). Despite the absence of charged S2-pocket interactions, this binding diversity highlights the unique profile of CID 46215462.

##### 3.2.4.6 Acetaminophen–Negative Control

Lastly, although acetaminophen had a favorable docking score (−5.71 kcal/mol), interaction matrix analysis revealed a complete absence of sustained interactions with pocket residues during MD (Figure 8E), correlating with loss of stable positioning within the active site. These results strongly underscore the critical need for MD in validating docking predictions, as previously reported,^71^ as it provides essential insights into the dynamic stability and binding site adaptability.

### 3.3 Membrane Permeation Studies

#### 3.3.1 Free Energy Profiles of Ligand Permeation across the Bilayer

The primary role of DPP-4*i* in glucose homeostasis is to potentiate the action of incretin hormones. Orally administered drugs depend on protecting incretins from degradation by DPP-4 anchored in the enterocyte brush border membrane and achieving optimal bioavailability for systemic impact.^72^ Effective diffusion across enterocyte membranes is essential, as suboptimal permeability compromises both local and systemic action.^73,74^ Accordingly, we integrated free energy determination into our virtual workflow, a critical parameter rarely evaluated in standard computational approaches despite its significant impact on the study of oral bioavailability.

We investigate absorption potential using umbrella sampling simulations with an enterocyte membrane lipid bilayer model (Figure 9A). This method calculates free energy as the ligand is gradually pulled from the bilayer center (0 Å, *Z*-axis) to the aqueous phase (35 Å, *Z*-axis) (Supporting Figure S2). Deep energy minima indicate strong stability within the membrane, while broad, extended minima suggest greater lateral mobility and favorably lipid interactions.^44^

**Figure 9:**
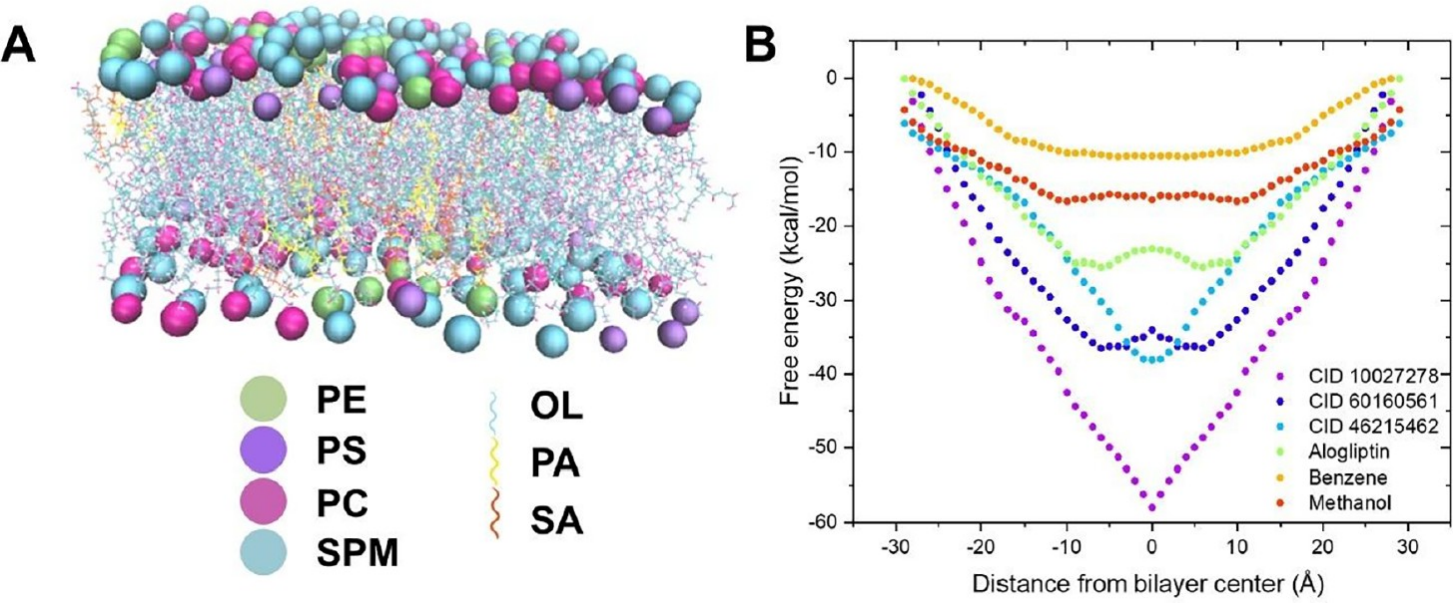
Representation of a model enterocyte lipid bilayer and the energetics of compound permeation through enterocyte membranes. (A) Representative view of the simulated lipid bilayer. Membrane was constructed with PACKMOL-Memgen, composed of phospholipids and sphingomyelin in molar ratios of DOPC: DOPE: PSM: DOPS (5:3:1:1). Colored circles represent the phospholipid heads to distinguish each type: phosphatidylcholine (PC, magenta), phosphatidylethanolamine (PE, violet), sphingomyelin (SM, cyan), and phosphatidylserine (PS, lime). The lipid tails include oleoyl (OL, 18:1, cyan), palmitoyl (PA, 16:0, yellow), and saturated fatty acid (SA, orange) chains. (B) Permeation free energy (PMF) profiles across the enterocyte bilayer were calculated for the compounds CID 46215462, CID 60160561, CID 10027278, alogliptin, methanol, and benzene using the Umbrella Sampling technique in AMBER. The *X*-axis represents the position along the reaction coordinate (*Z*-axis), where 0 Å corresponds to the center of the bilayer. Negative and positive values indicate the distance to the aqueous phases. The *Y*-axis shows the free energy (Δ*G*(*z*)) in kcal/mol.

CID 10027278 (magenta dotted line) and CID 46215462 (blue dotted line) showed similar permeation profiles with deep, narrow minima at the lipid bilayer center. CID 10027278 had the lowest free energy minimum (−56.19 kcal/mol), whereas CID 46215462 had a less negative value (−38.06 kcal/mol). Conversely, CID 60160561 (purple dotted line) and alogliptin (green dotted line) displayed distinct permeation profiles characterized by broad, extended energy minima. The minimum for CID 60160561 occurred between *z* = 0 and −5 Å (−36.22 to −35.21 kcal/mol). In comparison, Alogliptin showed higher energy at the bilayer center (∼−23.11 kcal/mol) with energy decreasing as the compound moved toward the interior of the monolayers (between 0 and ± 10 Å). Control compounds benzene (yellow dotted line; −10.48 kcal/mol) and methanol (orange dotted line; −16.46 kcal/mol) showed lower energy barriers, consistent with their reduced membrane affinity (Figure 9B).

The distinct free energy landscapes suggest different permeation mechanisms. Compounds with deep, narrow minima (CID 10027278, CID 46215462) show strong hydrophobic membrane affinity but may face kinetic barriers to complete passage. In contrast, those with broad, extended minima (CID 60160561, alogliptin) exhibit greater flexibility that could facilitate efficient membrane transit. The depth-to-breadth ratio of the energy minimum appears to be a key determinant of permeation efficiency.

These computational analyses provide valuable insights into the permeation profiles of DPP-4*i* candidates across the enterocyte plasma membrane. The umbrella sampling method enabled us to differentiate the permeability of compounds based on their membrane-interaction patterns, serving as an early stage filtering tool to prioritize them by favorable passive transport properties. However, *in vitro* studies remain essential to confirm these actual permeation rates and bioavailability.

#### 3.3.2 Spontaneous Ligand Behavior in An Enterocyte Membrane

To understand the spontaneous diffusion behavior, 200 ns MD simulations were conducted with 15 copies for each compound positioned in the aqueous phase outside an enterocyte bilipid bilayer (Figure 10A and Supporting Movies S4, S5, S6, S7). After 200 ns, all DPP-4*i* candidates and alogliptin control interacted with the membrane lipid head groups but partially diffused toward the center (Figure 10B–10E).

**Figure 10:**
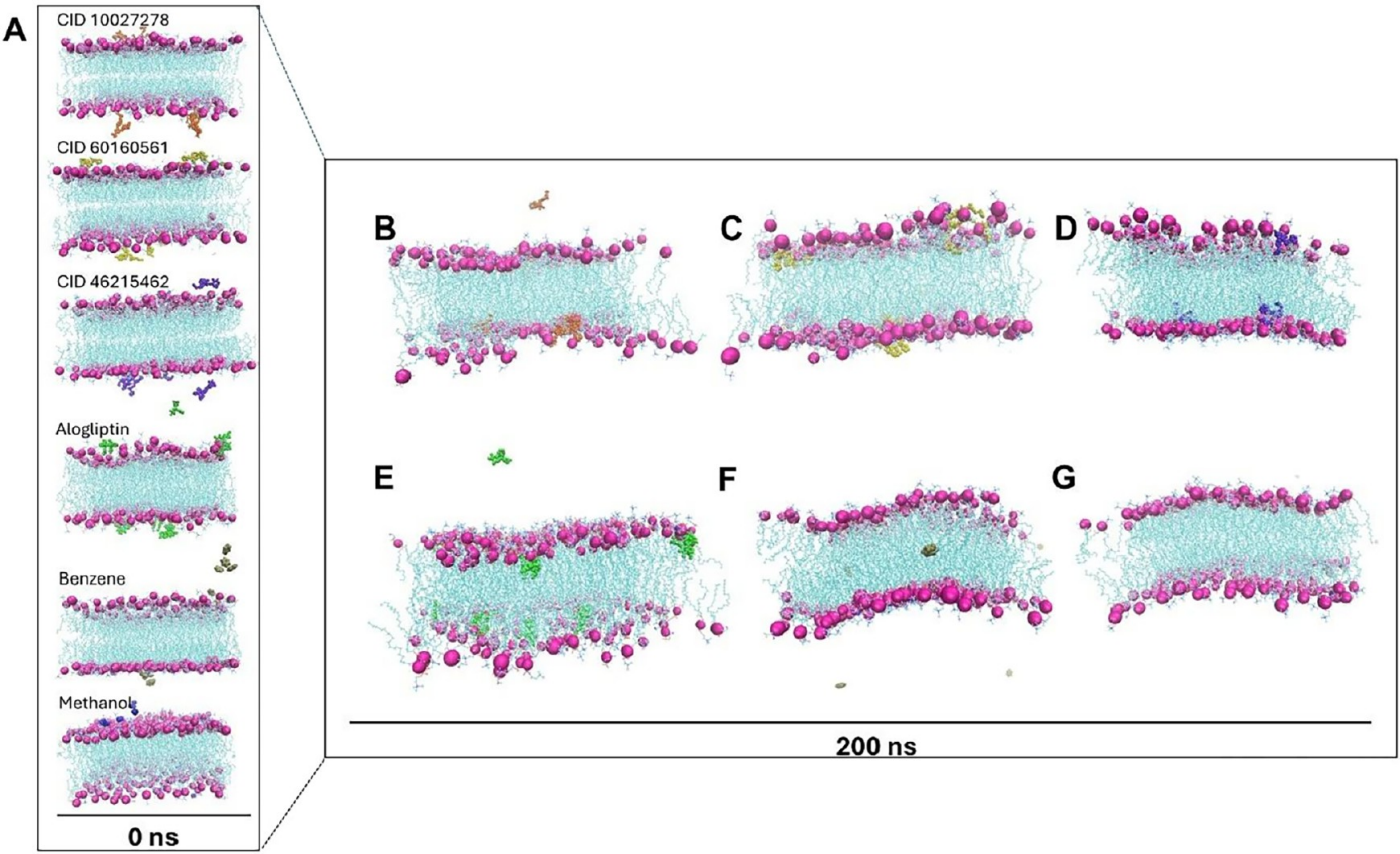
Distribution and spontaneous permeation of compounds in an enterocyte model lipid bilayer during 200 ns of molecular dynamics simulations. The lipid bilayer, composed of DOPC:DOPE:PSM:DOPS (5:3:1:1), was simulated with the compounds positioned without restrictions in the adjacent aqueous phase. (A) Shows the initial system configurations at 0 ns. Enlarged views of permeation into the bilayer at 200 ns of simulation are presented for (B) CID 10027278, (C) CID 60160561, (D) CID 46215462, (E) alogliptin, (F) benzene, and (G) methanol.

Quantitative contact analysis revealed differences in the magnitude and stability of the interactions between the compounds and membrane atoms (Supporting Figure S3). Progressive association with hydrophobic membrane components was observed (Supporting Figure S3A), with stable interactions throughout the simulation (Supporting Figure S3B). All compounds maintained contact constant with both upper and lower membrane leaflets (Supporting Figure S3C).

Control molecules demonstrated the fundamental impact of size and polarity in membrane interactions and permeation. Benzene, a small nonpolar molecule (MW: 78.11 g/mol), formed stable contacts during MD, quickly diffused toward the bilayer center, and successfully traversed the membrane (Figure 10F and Supporting Movie S8). Methanol, a small polar molecule (MW: 32.04 g/mol), exhibited few unstable interactions and rapidly transitioned between the bilayer and aqueous interfaces (Figure 10G and Supporting Movie S9), consistent with high permeability and low membrane retention. Based on these behaviors, the substantially higher molecular weights of the DPP-4*i* candidates (CID 10027278:460.4 g/mol, CID 6016051:576.1 g/mol, CID 46215462:506.6 g/mol) could explain the limited transmembrane passage.

Position analysis along the *z*-axis revealed distinct penetration behaviors (Supporting Figure S3D). CID 10027278 demonstrated a moderate insertion depth, greater than that of alogliptin, but remained primarily at outer surface lipids. Limited penetration despite a high thermodynamic affinity for the membrane core demonstrates that a deep energy minimum can create kinetic barriers, leading to surface trapping rather than rapid permeation. CID 60160561 initially penetrated but showed decreased insertion depth toward the end of the simulation, reflecting the challenge of larger molecules in maintaining a stable membrane position. It will be interesting to evaluate this magnitude in further studies, even if its ultimate passage was not observed within 200 ns MD. CID 46215462, in turn, exhibited the least internalization, remaining predominantly at the polar interface. Meanwhile, positional analysis of the control molecules validated the size-dependent effects.

Results from MD simulations validate our protocol for simulating permeation, successfully tracking molecular movement across enterocyte membranes without the need for external forces. Findings revealed an effective compound-membrane association while demonstrating that molecular size creates kinetic barriers that prevent complete transmembrane passage within the simulation time frame. However, given the complexity of permeation, simulation times exceeding 200 ns may be necessary for larger compounds to achieve complete insertion equilibrium.

These pioneering *in silico* analyses provide fundamental, cost-effective insights in the early stages of drug discovery and can considerably support candidate drug selection. Based on these promising results, we propose integrating this MD protocol into standard computational workflows, as it supports the identification of compounds with inherent bioavailability limitations before extensive optimization efforts, thus addressing one of the significant obstacles in oral drug development. Building upon the solid foundation of favorable characteristics established here, further *in vitro* and *in vivo* assays will be crucial to evaluate the complex processes of intestinal absorption of candidates.

## 4 Conclusions

This study demonstrates the strategic value of intelligent compound curation for virtual drug discovery. We explored the search for potential DPP-4*i*, considering only the ∼30,000 carefully selected bioactive compounds from natural sources deposited in the public database PubChem. Although it represents a fundamental departure from traditional high-throughput screening approaches, this workflow concentrates computational resources on molecules with verified therapeutic potential, rather than dispersing efforts across chemically diverse collections.

Using Uni-Dock software allowed us to both accelerate the docking process, significantly reducing computational time, and explore a broader chemical space in the search for drug candidates. The docking analysis identified 582 compounds with higher binding scores than those of reference drugs. Of these, eight compounds were subsequently validated through MD, confirming their improved binding energies. Following data set refinement using energy distribution as a primary criterion, we identified three lead candidates: OSU-03012, EPZ005687, and bemcentinib. These compounds maintained stable multisubsite binding patterns—S1, S2, S1′, S2′, or extensive S2 subsites—throughout 100 ns of simulation, as well as the residues adjacent to the pocket, suggesting potency mechanisms that distinguish them from current therapeutic options. The identification of compounds with proven therapeutic profiles offers translational opportunities and strategic advantages in diabetes drug development, as their clinical safety profiles can accelerate development timelines and reduce regulatory risks compared to novel chemical entities.

Moreover, our virtual workflow highlights the integration of membrane permeability predictions with specific lipid composition. Permeability simulations predicted partial penetration of all three lead compounds into the simulated membrane. The favorable energetic profiles support their potential for diffusion, which could favor subsequent DPP-4 inhibitory action in the intestine.

The successful identification of natural compounds with both potent target affinity and favorable membrane permeability validates our central hypothesis that intelligent screening strategies can simultaneously address efficacy and safety concerns in the development of diabetes drugs. These findings provide a roadmap for future virtual screening campaigns targeting complex therapeutic challenges where conventional synthetic compounds have reached limitations. Further *in vitro* investigation of the identified compounds will provide valuable insights into their therapeutic potential and mechanisms of action.

## Supplementary Material





















## Data Availability

All data supporting the conclusions of this paper are available within the manuscript and/or its Supporting Information.

## Notes

Molecular docking scores of compounds at the lowest energy percentile (Table S1); docking score, binding free energy, and properties of the compounds subjected to molecular dynamics simulation (Table S2); contribution of polar and nonpolar termini to ligand binding with DPP-4, calculated via MM-GBSA decomposition (Figure S1); representation of the initial and final pushing positions for the ligands across the lipid bilayer (Figure S2); analysis of interactions of compounds with a simulated enterocyte membrane (Figure S3) (PDF)
The trajectory of dipeptidyl peptidase-4 free is available as (Video S1) (MP4)
The trajectory of the complex between CID 10027278 and dipeptidyl peptidase-4 is available as (Video S2) (MP4)
The trajectory of the complex between acetaminophen and dipeptidyl peptidase-4 is available as (Video S3) (MP4)
Trajectories showing the spontaneous movement of CID 10027278 (Video S4) (MP4)
Trajectories showing the spontaneous movement of CID 60160561 (Video S5) (MP4)
Trajectories showing the spontaneous movement of CID 46215462 (Video S6) (MP4)
Alogliptin through the membrane (Video S7) (MP4)
Depicts the trajectory of benzene diffusing through the membrane (Video S8) (MP4)
Depicts the trajectory of methanol diffusing through the membrane (Video S9) (MP4)

## Abbreviations

DM: diabetes mellitus
DPP-4: dipeptidyl peptidase-4
DPP-4*i*: DPP-4 inhibitor
T2DM: type 2 diabetes
FDA: food and Drug Administration
GIP: glucose-dependent insulinotropic polypeptide
GLP-1: glucagon-like peptide-1
GPUs: Graphics processing units
HbA1c: Glycosylated hemoglobin
MD: Molecular dynamics
MM/GBSA: Molecular Mechanics with Generalized Born and Surface-Area
RMSD: Root-Mean-Square Deviation
RMSF: Root-Mean-Square Fluctuation.
