# Supplementary material for: GPU-Accelerated Virtual Screening and Molecular Dynamics Simulations for Identification of Novel DPP‑4 Inhibitors
Source: ACS Omega. 2026 Jan 21;11(4):5323–38. doi: 10.1021/acsomega.5c08231 (PMC12878728; doi:10.1021/acsomega.5c08231)
Supplement: Supplementary file 1 [file ao5c08231_si_001.pdf]

Supporting Information for:

GPU-Accelerated Virtual Screening and Molecular Dynamics Simulations for the Identification of Novel DPP-4 Inhibitors

Nathaly Vasquez-Martínez[a], Jonathan Trapala[b], Laura L. Álvarez-Añorve[a], Rodolfo Aáron Lizárraga Valadez[a], Martín González-Andrade[a]*, and Alejandro Sosa-Peinado[a]*

August 2025

[a]*Departamento de Bioquímica, Facultad de Medicina, Universidad Nacional Autónoma de México, Ciudad de México, CP. 04510, México*

[b]*Departamento de Ingeniería Celular y Biocatálisis, Instituto de Biotecnología, Universidad Nacional Autónoma de México, Cuernavaca, 62210, México*

***Correspondence:**

Email: asosa@unam.mx

Email: martin@bq.unam.mx

Supporting Information Content

Table S1. Molecular docking scores of compounds at the lowest energy percentile.
We performed a virtual screening of 30,698 commercially available bioactive compounds, filtered from the PubChem database, using UniDock. The table shows 0.6% of compounds in the database with the lowest binding energies (kcal/mol).

N°	Identification	Type	Score docking (kcal/mol)	CID	Name of the Compound
1	21998	NP-bμM	-12.08	135524769	4-(10,15,20-Triphenylporphyrin-5-yl)phenol
2	13621	NP-bμM	-11.841	158365	Win 64821
3	11820	NP-bμM	-11.831	60775	Ro-24-4736
4	12136	NP-bμM	-11.781	70186	CHEMBL345046
5	2826	NP-bnM	-11.224	42611190	PPTN
6	8961	NP-bμM	-11.184	138757257	CHEMBL5201896
7	3443	NP-bμM	-11.115	60160561	EPZ005687
8	19308	NP-bμM	-11.006	9916195	SR 144190
9	13070	NP-bμM	-10.896	104903	Tirilazad
10	21746	NP-bμM	-10.894	132280527	CHEMBL4454538
11	8962	NP-bμM	-10.858	138757260	CHEMBL5200323
12	4967	NP-bμM	-10.83	9917862	Dirlotapide 8Slentrol
13	4187	NP-bμM	-10.798	132916	Pranazepide
14	7081	NP-bμM	-10.795	10127889	CHEMBL193084
15	1984	NP-bμM	-10.783	24762176	CHEMBL1213559
16	4953	NP-bμM	-10.66	9913629	LB-30057
17	17798	NP-bμM	-10.568	4695397	Phenylglycine-01
18	8910	NP-bμM	-10.555	136244897	CHEMBL2322213
19	7395	NP-bμM	-10.554	12151052	874948-59-1
20	8209	NP-bμM	-10.537	56965952	CHEMBL2177836
21	4929	NP-bμM	-10.526	9894349	TAK-637
22	19284	NP-bμM	-10.524	9907241	CHEMBL28853
23	17659	NP-bμM	-10.522	3974222	MLS000559184
24	22050	NP-bμM	-10.514	135899519	CHEMBL461940
25	7813	NP-bμM	-10.509	44265442	CHEMBL268737
26	14867	NP-bμM	-10.494	442985	Solasodine
27	19955	NP-bμM	-10.468	11289099	Phenylethyl beta-D-glucopyranoside
28	3680	NP-bμM	-10.454	4257	Mosapramine
29	8532	NP-bμM	-10.442	122188889	CHEMBL3613293
30	8963	NP-bμM	-10.44	138757242	CHEMBL5178855
31	17483	NP-bμM	-10.422	3350112	CHEMBL334635
32	8304	NP-bμM	-10.403	71517117	Fradcarbazole A
33	20642	NP-bμM	-10.385	16742146	Unii-DQ4lkh74UY (JNJ-38893777)
34	19205	NP-bμM	-10.379	9875424	Bms453
35	1672	NP-bnM	-10.363	151171	Conivaptan
36	5708	NP-bμM	-10.363	156124857	MRTX1133
37	2172	NP-bμM	-10.359	25105705	CHEMBL1213068
38	21370	NP-bμM	-10.355	54692153	CHEMBL32926
39	105	NP-bnM	-10.346	57030	Brequinar
40	20979	NP-bμM	-10.346	25234918	Gdc-0834
41	5540	NP-bμM	-10.332	72163100	Atogepant
42	7118	NP-bμM	-10.33	10311947	CHEMBL392867
43	750	NP-bnM	-10.33	24822449	CHEMBL410473
44	9112	NP-bμM	-10.302	155920430	CHEMBL4755343

N°	Identification	Type	Score docking (kcal/mol)	CID	Name of the Compound
45	7077	NP-bµM	-10.286	10127629	CHEMBL372659
46	843	NP-bµM	-10.279	72812	Bathophenanthroline
47	14979	NP-bµM	-10.279	448062	Zk-806450
48	16370	NP-bµM	-10.274	1257244	MLS000583768
49	4958	NP-bµM	-10.272	9917705	CHEMBL159418
50	2822	NP-bnM	-10.262	25262792	Vistusertib
51	1472	NP-bµM	-10.26	65149	Bathocuproin
52	7255	NP-bµM	-10.255	11190026	CHEMBL426597
53	2482	NP-bnM	-10.254	10022859	CHEMBL281274
54	13508	NP-bµM	-10.245	148195	Lonafarnib
55	3541	NP-bµM	-10.227	156125086	US11453683, Example 281
56	1877	NP-bµM	-10.22	10579727	CHEMBL105569
57	20092	NP-bµM	-10.214	11494412	Tivantinib
58	19326	NP-bµM	-10.21	9939865	LY 517717
59	13017	NP-bµM	-10.207	99474	DIOSGENIN
60	7250	NP-bµM	-10.204	11166601	CHEMBL369324
61	5519	NP-bµM	-10.2	68748835	Ubrogepant
62	19077	NP-bµM	-10.191	9829523	Midostaurin
63	5747	NP-bµM	-10.187	219077	Osanetant
64	711	NP-bnM	-10.16	10483775	CHEMBL3127463
65	2658	NP-bnM	-10.16	11656518	Raf-265
66	20838	NP-bµM	-10.159	24771429	639509-22-1
67	1531	NP-bµM	-10.159	107715	Dihydroergocristine
68	7299	NP-bµM	-10.142	11418865	CHEMBL178754
69	21424	NP-bµM	-10.138	56945296	3-Hydroxy-2-[3-hydroxy-4-(pyrrolidin-1-yl)phenyl]benzo[h]chromen-4-one
70	2189	NP-bµM	-10.137	25174101	pyrazolourea, 1b
71	16628	NP-bµM	-10.13	2063288	78660-92-1
72	2100	NP-bnM	-10.125	5311201	L-365260
73	3115	NP-bµM	-10.119	2883607	Trisindoline
74	17046	NP-bµM	-10.118	2853195	GNF-Pf-4095
75	7105	NP-bµM	-10.113	10239716	CHEMBL371781
76	3969	NP-bµM	-10.101	59751	Rac-Devazepide
77	5416	NP-bµM	-10.1	46215462	Bemcentinib
78	1626	NP-bµM	-10.095	657356	diacylglycerol kinase inhibitor ii
79	12764	NP-bµM	-10.083	91766	Flufenoxuron
80	19125	NP-bµM	-10.077	9850972	CHEMBL114575
81	1741	NP-bµM	-10.071	5284329	Purmorphamine
82	4930	NP-bµM	-10.068	9891901	NGB 2904
83	1824	NP-bnM	-10.066	444031	Darifenacin
84	12880	NP-bµM	-10.064	94483	38103-06-9
85	8258	NP-bµM	-10.064	67452275	CHEMBL4546800
86	5361	NP-bµM	-10.062	25232541	MOR Partial Agonist NAQ
87	7310	NP-bµM	-10.06	11488329	PHELLIGRIDIN E
88	624	NP-bµM	-10.053	44475986	CHEMBL1213117
89	8918	NP-bµM	-10.051	136237391	CHEMBL2172756
90	4535	NP-bµM	-10.044	3038517	Sufugolix

N°	Identification	Type	Score docking (kcal/mol)	CID	Name of the Compound
91	7508	NP-bμM	-10.038	16094766	GP 2A
92	5168	NP-bμM	-10.032	11527495	Ibodutant
93	796	NP-bnM	-10.029	58466119	CHEMBL3394057
94	633	NP-bnM	-10.028	156125087	US11453683, Example 189
95	19085	NP-bμM	-10.024	9832179	Ro 28-2653
96	7253	NP-bμM	-10.018	11177309	CHEMBL175848
97	2416	NP-bnM	-10.016	9915041	Dpc 602
98	6495	NP-bμM	-10.008	104934	u-74389g
99	159	NP-bnM	-10.006	44475990	CHEMBL1213118
100	1066	NP-bμM	-10.006	53364540	KT109
101	787	NP-bμM	-10.006	11053	Violacein
102	17030	NP-bμM	-10.005	2841659	MLS000703710
103	3479	NP-bμM	-10	119081677	CHEMBL3629567
104	8303	NP-bμM	-9.998	71522029	CHEMBL2282461
105	8894	NP-bμM	-9.99	135976517	ANA598
106	7867	NP-bμM	-9.984	44369868	CHEMBL349577
107	17849	NP-bμM	-9.982	5096779	CHEMBL1472604
108	18999	NP-bμM	-9.979	9808655	u-83836e
109	12620	NP-bμM	-9.972	82343	Naugard 445
110	19452	NP-bμM	-9.97	10027278	OSU-03012
111	20267	NP-bμM	-9.967	12003241	Pluripotin
112	2765	NP-bμM	-9.965	10098	JERVINE
113	860	NP-bnM	-9.964	156125323	KRAS G12D inhibitor 1
114	7992	NP-bμM	-9.962	44574481	CHEMBL466095
115	1715	NP-bnM	-9.962	176167	Enzastaurin
116	2763	NP-bnM	-9.962	23653789	Serlopitant
117	1588	NP-bnM	-9.948	119342	4-Ttab, CD-367
118	861	NP-bnM	-9.932	156124706	US11453683, Example 179
119	859	NP-bnM	-9.895	156124936	CHEMBL4863339
120	2090	NP-bnM	-9.882	5311147	GV150013
121	1817	NP-bnM	-9.876	439005	CHEMBL372469
122	620	NP-bnM	-9.865	56927660	APY0201
123	149	NP-bnM	-9.859	24767923	CHEMBL1213152
124	154	NP-bnM	-9.849	25097993	CHEMBL1213084
125	826	NP-bnM	-9.835	129188688	DH376
126	625	NP-bnM	-9.829	118557502	Gsk-3145095
127	3002	NP-bnM	-9.814	91050674	CHEMBL4450227
128	168	NP-bnM	-9.796	123132216	CHEMBL4080018
129	2956	NP-bnM	-9.776	67960061	CHEMBL3393706
130	2795	NP-bnM	-9.766	25066467	Rebastinib/DCC-2036
131	93	NP-bnM	-9.759	5074	Ritanserin
132	2718	NP-bnM	-9.757	16049791	Vialinin B
133	2157	NP-bnM	-9.753	5480230	Norbinaltorphimine
134	2519	NP-bnM	-9.745	10252734	GP 1A
135	3165	NP-bnM	-9.736	57345941	Belizatinib
136	1622	NP-bnM	-9.735	126565	Lestaurtinib
137	997	NP-bnM	-9.727	3822	Ketanserin
138	2849	NP-bnM	-9.709	44593666	Bms-694153

N°	Identification	Type	Score docking (kcal/mol)	CID	Name of the Compound
139	2065	NP-bnM	-9.656	5281600	Amentoflavone
140	634	NP-bnM	-9.637	156125195	CHEMBL4867851
141	834	NP-bnM	-9.628	132156530	CHEMBL4595328
142	2841	NP-bnM	-9.613	44454750	CORT-108297
143	2803	NP-bnM	-9.613	25141092	Entrectinib
144	1621	NP-bnM	-9.601	126437	Furamidine
145	626	NP-bnM	-9.588	134134891	CHEMBL3907029
146	509	NP-bnM	-9.583	443590	Telomestatin
147	2479	NP-bnM	-9.578	10004545	AMG-076 free base
148	1199	NP-bnM	-9.573	8223	ERGOTAMINE
149	839	NP-bnM	-9.572	134150995	CHEMBL3966490
150	787	NP-bnM	-9.57	53248722	3-anthracen-9-yl-5-(4-methoxy-phenyl)-4,5-dihydro-1hpyrazole
151	2527	NP-bnM	-9.57	10296883	Sotrastaurin
152	167	NP-bnM	-9.567	121328278	Linrodostat
153	2182	NP-bnM	-9.551	6419753	CK1 Inhibitor
154	2085	NP-bnM	-9.546	5311192	IRL-2500
155	2730	NP-bnM	-9.541	16734800	LXR-623
156	2720	NP-bnM	-9.535	16124208	TAK-901
157	2757	NP-bnM	-9.534	23649154	Tegobuvir
158	2205	NP-bnM	-9.518	6451149	Netupitant
159	611	NP-bnM	-9.515	644241	Nilotinib
160	937	NP-bnM	-9.51	2894	CHEMBL97698
161	2876	NP-bnM	-9.501	49806720	Alectinib
162	2448	NP-bnM	-9.501	9937291	Olodanrigan
163	2687	NP-bnM	-9.5	11960895	TCS 1102
164	1550	NP-bnM	-9.489	104974	Saredutant
165	2253	NP-bnM	-9.486	6918602	Relacatib
166	697	NP-bnM	-9.484	10237492	CHEMBL193316
167	1701	NP-bnM	-9.483	164456	Cd 2019
168	674	NP-bnM	-9.481	9822488	CHEMBL208824
169	1753	NP-bnM	-9.471	213046	Lurasidone
170	332	NP-bnM	-9.469	3081355	SR144528
171	2436	NP-bnM	-9.465	9927531	BIBR 1532
172	2874	NP-bnM	-9.464	46911863	JNJ-38877605
173	2318	NP-bnM	-9.45	9826034	GNTI
174	1702	NP-bnM	-9.444	170364	Farglitazar
175	1840	NP-bnM	-9.444	443375	DEVAZEPIDE
176	2117	NP-bnM	-9.44	5310960	MRE 3008-F20
177	123	NP-bnM	-9.428	1547484	Cinnarizine
178	583	NP-bnM	-9.425	24762166	Purfalcamine
179	2904	NP-bnM	-9.422	54576299	Altiratinib
180	2076	NP-bnM	-9.418	5289501	Arotinoid acid
181	2807	NP-bnM	-9.416	25145656	capmatinib
182	145	NP-bnM	-9.413	15485192	CHEMBL219585
183	1968	NP-bnM	-9.41	3002977	Maraviroc
184	2938	NP-bnM	-9.409	57519544	Wnt-C59
185	2932	NP-bnM	-9.405	56944144	Lemborexant
186	385	NP-bnM	-9.386	24873449	Dagrocorat

N°	Identification	Type	Score docking (kcal/mol)	CID	Name of the Compound
187	2298	NP-bnM	-9.376	9805452	SoRI-9409
188	2350	NP-bnM	-9.37	9853053	Lomitapide
189	2419	NP-bnM	-9.683	9913629	LB-30057
**Positive control (FDA-approved inhibitor)**					
1	26812	bnM	-9.284	10096344	Linagliptin
2	26813	bnM	-8.156	4369359	Sitagliptin
3	26814	bnM	-7.257	6918537	Vildagliptin
4	26815	bnM	-6.875	11450633	Alogliptin
5	26816	bnM	-6.42	11243969	Saxagliptin
**Negative control**					
1	26817		-8.4959	11983	Acetaminophen

Abbreviations

CID PubChem compound identifier

bμM Bioactive compounds in the micromolar range

bnM Bioactive compounds in the nanomolar range

Table S2. Docking score, binding free energy, and properties of the compounds subjected to molecular dynamics simulation. Docking score, binding free energy, and properties of the compounds subjected to molecular dynamics simulation. After molecular docking of the database, we selected the compounds following the inclusion criteria described in Materials and Methods. The calculated docking score and binding free energy (kcal/mol) are listed, along with the activity, therapeutic potential, and chemical classification described for these compounds. The results obtained for the reference drugs and the negative control are included.

CID	Name of the Compound	Score docking (kcal/mol)	Binding Affinity (kcal/mol)	Activity	Therapeutic potential
60160561	EPZ005687	-11.115	-58.1841 ± 3.4655	Inhibitor of the lysine methyltranferase EZH2	Antineoplastic activity
10027278	OSU-03012	-9.97	-51.7302 ± 5.379	Phosphoinositide-dependent kinase 1 (PDK1) inhibitor	Antineoplastic agent and an apoptosis inducer
46215462	Bemcentinib	-10.1	-44.05113 ± 6.57403	Inhibitor of the AXL receptor tyrosine kinase (UFO)	Antineoplastic activity
10004545	AMG-076 free base	-9.578	-44.4815	Melanin-concentrating hormone receptors (MCHR) antagonist	Metabolic-cardiovascular diseases; treatment of obesity
10296883	Sotrastaurin	-9.57	-43.3379	Protein kinase C (PCK)-selective inhibitor	Immunosuppressive and antineoplastic activities
5074	Ritanserin	-9.759	-41.4973	Seratonin (5-hydroxytryptamine, 5-HT) antagonist	Anxiety, depression and schizophrenia
3038517	Sufugolix	-10.044	-40.8797	Antagonist at the human receptor (hGnRHR)	Use/treatment in endometriosis and uterine fibroids
25066467	Rebastinib	-9.766	-39.6757	Tie2 tyrosine kinase receptor inhibitor	Antineoplastic activity
9916195	SR 144190	-11.006	37.5809	Tachykinin NK2 Receptor Antagonists	Nervous system diseases
126437	Furamidine	-9.601	-35.4534	Protein arginine methyltransferase 1 (PRMT1) inhibitor	Chronic Obstructive Pulmonary Disease. Antiparasitic activity
24873449	Dagrocorat	-9.386	-33.924	Agonist of the glucocorticoid receptor	Rheumatoid arthritis
11656518	Raf-265	-10.16	-33.906	B-Raf/VEGFR-2 Inhibitor	Antineoplastic activity
57030	Brequinar	-10.346	-33.8492	Dihydroorotate dehydrogenase (quinone)] inhibitor	Antineoplastic activity
99474	Diosgenin*	-10.207	-32.3998	Anti-inflammatory and neuroprotective effects	Apoptosis inducer, antiviral agent, antineoplastic agent and a metabolite
25234918	GDC-0834	-10.346	-31.9037	Bruton´s tyrosine kinase. (BTK) inhibitor	Anti-arthritis
9937291	Olodanrigan	-9.501	-31.8737	Angiotensin II type 2 receptor antagonist	Painful Diabetic Neuropathy
23653789	Serlopitant	-9.962	-30.5466	Neurokinin-1 (NK-1) receptor antagonists	Treatment of Prurigo Nodularis
46911863	JNJ-38877605	-9.464	-30.5223	c-Met tyrosine kinase inhibitor	Antineoplastic activity

CID	Name of the Compound	Score docking (kcal/mol)	Binding Affinitity (kcal/mol)	Activity	Therapeutic potential
5281600	Amentoflavone**	-9.656	-29.8508	Cathepsin B inhibitor. GABA(A) negative modulator	Anti-inflammatory, antioxidative, anti-viral, anti-tumor, anti-radiation, anti-fungal, antibacterial activity
104903	Tirilazad	-10.896	-29.1563	Iron-dependent lipid peroxidation inhibitor	Neuroprotective agents in the treatment of spinal cord injury
126565	Lestaurtinib	-9.735	-28.7119	Tyrosine kinase inhibitor	Antineoplastic activity
107715	Dihydroergocristine	-10.159	-28.7948	Adrenergic antagonist and a vasodilator agent	Symptomatic treatment of mental deterioration associated with cerebrovascular insufficiency and in peripheral vascular disease.
23649154	Tegobuvir	-9.534	-28.0693	Inhibitor of the HCV NS5B polymerase	Treatment of Hepatitis C chronic
60775	Ro-24-4736	-11.831	-22.2189	Platelet activating factor (PAF) antagonist	Asthma
11053	Violacein**	-10.006	-20.5674	Chromogenic bacterial secondary metabolites	Antibacterial, antifungal, antiprotozoan, and anticancer properties
16124208	TAK-901	-9.535	-18.3595	Aurora B Serine/Threonine Ki nase Inhibitor	Antineoplastic activity
11527495	Ibodutant	-10.032	-16.0707	Neurokinin-2 (NK-2) receptor antagonist.	Irritable Bowel Syndrome With Diarrhea.
44454750	Cort 108297	-9.613	-5.7873	Glucocorticoid Receptor Antagonist	Acute Stress
**Positive control (FDA-approved inhibitor)**					
10096344	Linagliptin	-9.284	-38.4077	Dipeptidyl-peptidase IV) inhibitor	Orally active hypoglycemic (anti-diabetic drug)
11450633	Alogliptin	-6.875	-34.6603 ± 4.8676	Dipeptidyl-peptidase IV) inhibitor	Orally active hypoglycemic (anti-diabetic drug)
6918537	Vildagliptin	-7.257	-31.5602	Dipeptidyl-peptidase IV) inhibitor	Orally active hypoglycemic (anti-diabetic drug)
4369359	Sitagliptin	-8.156	-28.1519	Dipeptidyl-peptidase IV) inhibitor	Orally active hypoglycemic (anti-diabetic drug)
11243969	Saxagliptin	-6.42	-24.8297	Dipeptidyl-peptidase IV) inhibitor	Orally active hypoglycemic (anti-diabetic drug)
**Negative control**					

CID	Name of the Compound	Score docking (kcal/mol)	Binding Affinitity (kcal/mol)	Activity	Therapeutic potential
11983	Acetaminophen	-5.71	-9.1541 ± 4.90216	Cyclooxygenase 2 inhibitor, a cyclooxygenase 1 inhibitor, a non-narcotic analgesic, an antipyretic, a non-steroidal anti-inflammatory drug, a cyclooxygenase 3 inhibitor	Analgesic

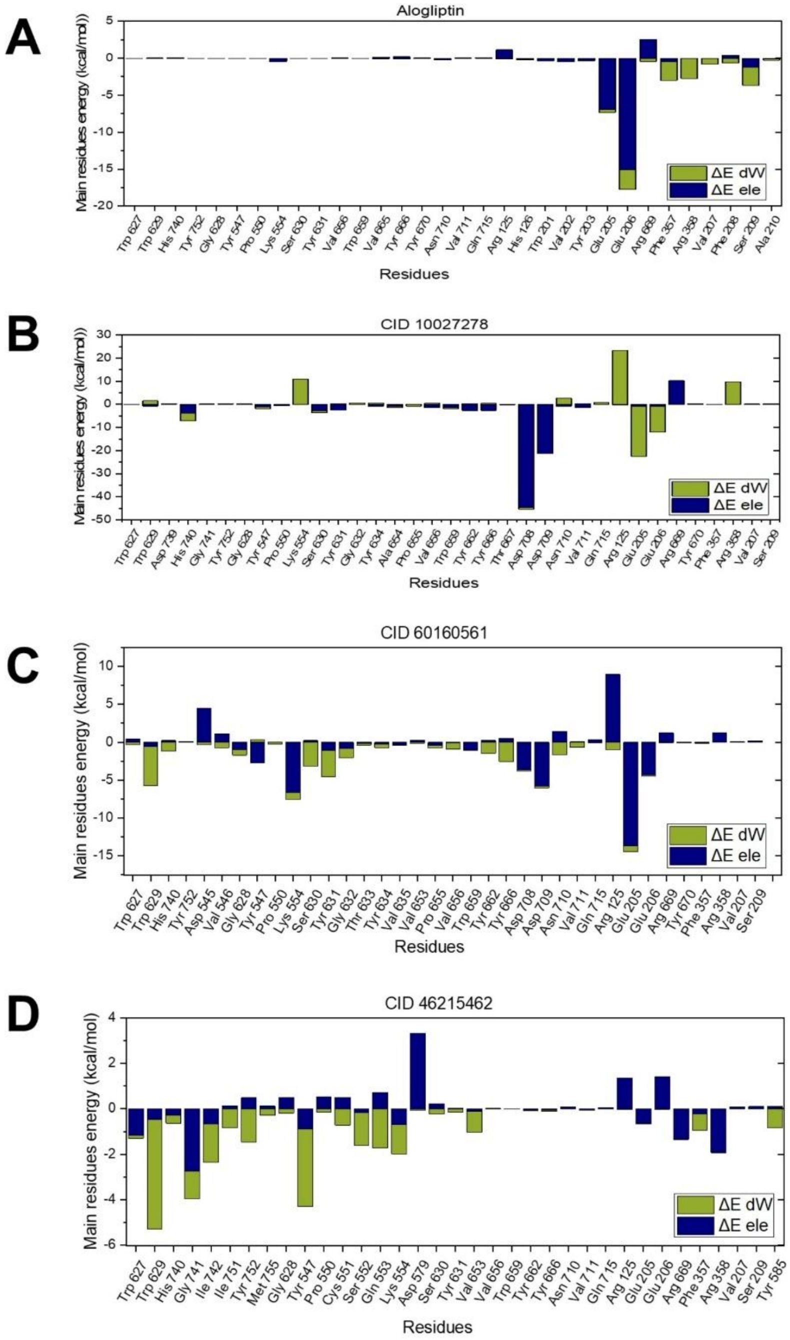

Figure S1. Contribution of polar and non-polar termini to ligand binding with DPP-4, calculated via MM-GBSA decomposition. The figures detail the energetic contributions of residues within the DPP-4 binding pocket, separating electrostatic (ΔG_{ele}) and van der Waals (Δ_{vdW}) interactions. Panels show results for A) Alogliptin, B) CID 10027278, C) CID 60160561, and D) CID 46215462 compounds.

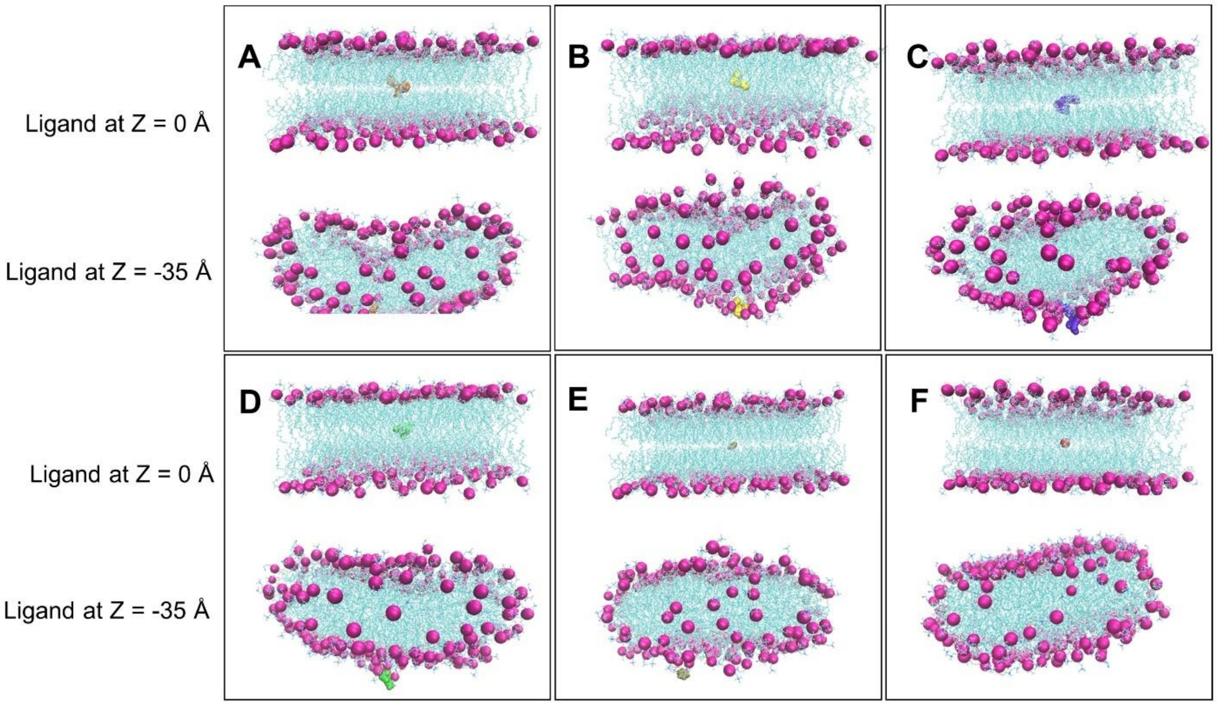

Figure S2. Representation of the initial and final pushing positions for the ligands across the lipid bilayer. Each panel shows two representative snapshots of the initial position at the center of the bilayer (Z = 0 Å, top) and the final position in the opposite aqueous phase (Z = -35 Å, bottom). Panels illustrate the results for A) CID 10027278, B) CID 60160561, C) CID 46215462, D) alogliptin, E) benzene, F) methanol. The ligand was gradually displaced along the Z axis by a harmonic restraint force, using an Umbrella Sampling simulation performed with AMBER.

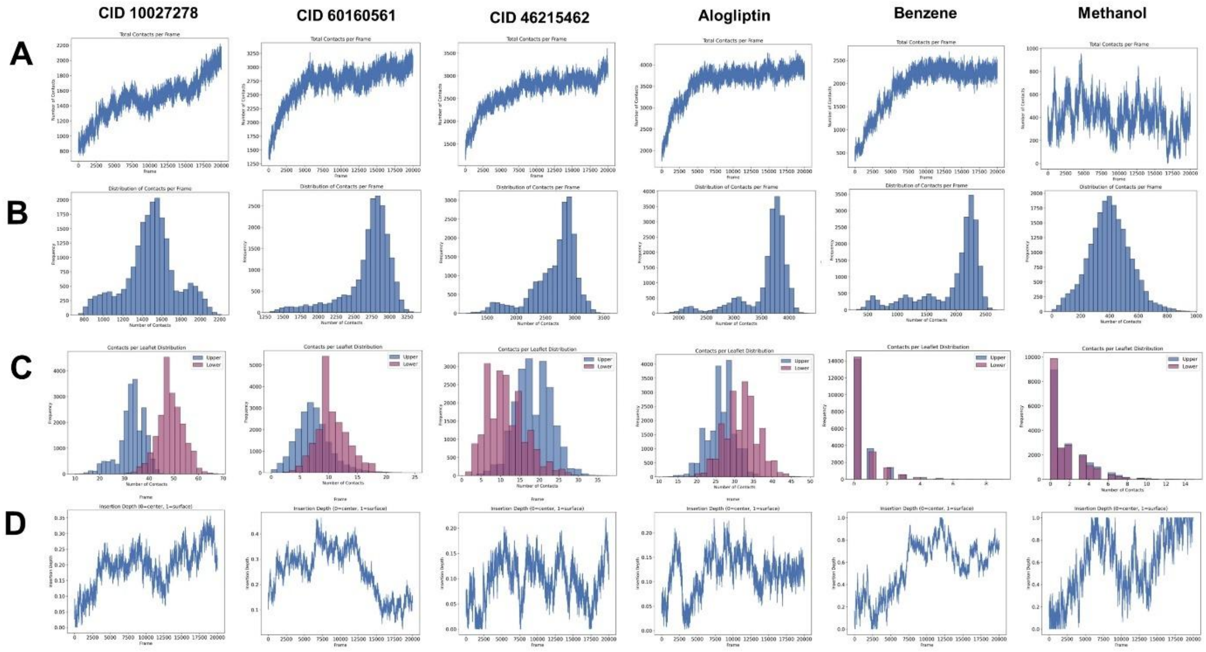

Figure S3. Analysis of interactions of compounds with a simulated enterocyte membrane. The figure presents the results of a 200 ns simulation for six different compounds (CID 10027278, CID 60160561, CID 46215462, alogliptin, benzene, and methanol) to study their spontaneous movement and permeation through an enterocyte membrane model. A) Shows "Total Contacts per Frame," representing the number of contacts formed between the atoms of the lipid bilayer and the atoms of each ligand over the 200 ns simulation. B) Illustrates the "Distribution of Contacts per Frame," indicating the frequency at which different numbers of contacts between the membrane and the ligands are observed. C) Presents the "Contacts per Ligand Distribution" on the membrane faces. "Upper" refers to contacts on the upper part of the membrane, while "Lower" indicates contacts on the lower part of the membrane, showing the frequency of these contacts on the external surfaces. D) Reflects the "Insertion Depth (Z-coord)," calculated from the center of mass of each ligand. On this Z-axis, a value of 0 corresponds to the center of the bilayer, and 1 represents the membrane surface. A value closer to 1 indicates that the ligands traversed the bilayer from one side to the other. In contrast, values closer to 0 suggest that the ligands moved from one side towards the center of the membrane, providing information on their permeation and localization within the bilayer.
